# An inter-city input-output database distinguishing firm ownership in the Greater China area during 2002–2017

**DOI:** 10.1038/s41597-025-04996-9

**Published:** 2025-05-01

**Authors:** Yafei Wang, Dingyi Xu, Heran Zheng, Ming Ye, Meichen Zhang, Qi He, Kun Cai, Dabo Guan, Shantong Li, Hong Ma, Bo Meng, Zhi Wang, Yang Wang, Lixiao Xu, Xianchun Xu, Peihao Yang, Qianhong Ouyang

**Affiliations:** 1 School of Statistics, Beijing Normal University, Beijing, 100875 China; 2 International Business School, Shanghai University of International Business and Economics, Shanghai, 201620 China; 3 The Bartlett School of Sustainable Construction, University College London, London, WC1E 7HB UK; 4 Yangtze IEDI, Nanjing University, Nanjing, 210023 China; 5 Research Institute for Global Value Chains, University of International Business and Economics, Beijing, 100029 China; 6 Department of Earth System Science, Tsinghua University, Beijing, 100084 China; 7 Department of Development Strategy and Regional Economy, Development Research Centre for the State Council, Beijing, 100010 China; 8 School of Economics and Management, Tsinghua University, Beijing, 100084 China; 9 Institute of Developing Economies, Japan External Trade Organization, Chiba, 261-8545 Japan; 10 The Collaborative Innovation Center for Emissions Trading System Co-constructed by the Province and Ministry, Wuhan, Hubei Province 430205 China; 11 Schar School of Policy and Government, George Mason University, 3351 Fairfax Drive, MSN 3B1, Arlington, VA 22201 USA; 12 Digital Economy Laboratory, University of International Business and Economics, Beijing, 100029 China; 13 School of Data Science, Zhejiang University of Finance and Economics, Hangzhou, 310018 China; 14 National School of Development, Peking University, Beijing, 100871 China

**Keywords:** Economics, Databases

## Abstract

Most multi-region input-output (MRIO) tables in China focus on provinces or urban agglomerations and ignore the tremendous geographical heterogeneities of economic activities across Chinese prefectural cities, where regional economic centres are usually located for domestic and global production. This paper constructs an inter-city input-output (IO) database with 42 sectors in the Greater China area. Compared with previous MRIO tables, it has three important features: (1) A complete coverage of Chinese cities, including 335 prefectural cities, four municipalities, and Hong Kong, Macao, and Taiwan; (2) Distinguishes three types of firm ownership for every city and sector for four benchmark years; (3) A novel data completion approach to reconcile all accessible micro-level data with city and provincial-level aggregate statistics, and effectively combining the bottom-up and top-down methods commonly used in the MRIO compilation literature. The database can be used in a diverse range of socioeconomic and interdisciplinary issues in the Greater China area at the city level. It also sheds light on other large economies to develop their own inter-city IO tables.

## Background & Summary

Given that China spans more than 9.6 million square kilometres, many of its highest-level administration divisions (e.g., provinces, autonomous regions, municipalities, and special administrative regions) are very large in their area and population. A large province comprises many cities with vast variations in their economic structures and development levels. For example, Guangdong, the province with highest gross domestic product (GDP) in China, contributes more than 10% of the GDP of China. Its GDP per capita and the human development index are both very high among China’s provinces. However, the GDP and other development indicators of its cities vary significantly. Guangzhou and Shen Zhen, the two largest cities among the 21 prefecture-level cities of Guangdong, jointly account for nearly half of the total GDP of the province. The nine cities of Guangdong in the Pearl River Delta (PRD), including Guangzhou and Shenzhen, contribute more than 80% of Guangdong’s total GDP, whereas the five least developed cities in the province only contribute less than 5% of its GDP^1^. The Gini coefficients estimated from GDP per capita show that the differences between-city in Guangdong, ranging from 0.33 to 0.42, which are much higher than those between-province from 0.03 to 0.22 during 2002–2017. To capture the intermediate and final product supply and demand linkages among these high diverse cities across the mainland of China, the large-scale high-resolution inter-city input-output (IO) table is needed to reflect detailed economic heterogeneities across cities.

In addition to the significant heterogeneity across cities within the mainland of China, the linkage of special administrative regions and Taiwan to the mainland of China is also noteworthy, as these regions differ substantially from mainland cities in terms of institution framework, structure of economy, as well as their roles in global value chains (GVC). For this reason, we also attempt to include these regions as a part of our IO tables so that to allow the analysis of the linkage between them and the mainland.

Hong Kong, Macao, and Taiwan can also be categorized into two groups based on the way they link the mainland of China to the international market. Hong Kong and Macao focused on the superior port system and aviation hub^2^, international financial and trade centres, and modern services. They belong to the Greater Bay Area (henceforth GBA), an integrated area of South China that includes nine cities of Guangdong Province in the Pearl River Delta, along with Hong Kong and Macao^3^. The GBA is one of China’s most dynamic urban agglomerations, characterized by its high degree of openness and dynamics of economic activities. In 2022, the GBA contributed more than 10% of China’s total national GDP (13.04 trillion CNY, around US $ 1.94 trillion)^4–6^, and it has long been recognized as one of the critical geographic areas to understand the linkage between China’s domestic supply chains and China’s participation in global production network. Therefore, incorporating Hong Kong and Macao into China’s city-level IO tables provides the international research community with a valuable database to study what roles the GBA could play in East Asia and the global economy.

Taiwan’s economic linkage to mainland China is somehow different from that of Hong Kong and Macao, as it relies more on foreign direct investment (FDI). Since the 1980s, Taiwan has maintained very close direct economic linkages with the mainland in addition to its indirect linkage to the mainland through Hong Kong and Macao, and it has been one of the major sources of FDI in the mainland of China. As of the end of 2020, Taiwan had invested a total of 70 billion US dollars, accounting for 3.1 per cent of total FDI in mainland China and it had established more than 117 thousand factories (11.2% of the total) in the mainland^7^. The key industrial linkage between mainland China and Taiwan is manufacturing. Taiwan has transferred its traditional labour-intensive manufacturing and electronic industries into many prefectural cities in the mainland such as Zhengzhou and Xi’an. Furthermore, Taiwan’s investment and manufacturing activities in the mainland are usually concentrated in export-oriented sectors, i.e., sectors that mainly participate in international market through vertical FDI and specialisation in the global supply chains^8^. To help the international research community capture the important roles of Taiwan in China’s domestic supply chains and the East Asian and global supply chains, we also integrate its IO tables into our inter-city IO database.

From the methodology perspective, the product-mix problem in sectoral/regional aggregation at different levels are the most crucial issue facing the IO communities. Compiling an IO table is usually based on the assumption of homogeneous products^9,10^. However, the product-mix problem in IO tables originates from firms in the same sector that produce different sets of products through heterogeneous technologies^11^. This aggregation issue can be more severe in multi-region input-output (MRIO) tables, which have been acknowledged as suitable databases to quantify the economic interdependence among industries in different economic regions^11^. MRIO databases have been developed at global^12–21^, national^22–27^, and subnational^28–31^ levels and applied to economic, social and environmental issues such as value-added trade^32,33^, employment^34^ and inequality^35^, as well as GHG emissions^36^ and carbon footprint estimation^37^. A variety of Chinese subnational MRIO tables have been constructed by different research groups^38–48^ and are widely used in the research communities^49–53^. Most previous subnational MRIOs for China use commodity sectors for their table construction^41,43–45,54^ and, therefore, cannot avoid the aggregation bias caused by the product-mix issue. Some studies have considered the heterogeneity of production technologies by different types of firms^55–57^. One stream of firm heterogeneity treatments is from the global production perspective, which separates processing and normal trading firms^58–61^. The other is from a domestic perspective, which distinguishes foreign-invested enterprises (FIEs) from Chinese-owned enterprises (COEs)^62,63^. A recent paper published further distinguishes FIEs into Hong Kong, Macao, and Taiwan (HMT) invested enterprises and other FIEs^8^ to capture special features of China’s FDI^64^. These efforts somewhat reduced the product-mix problem, but most of these studies were conducted at the national or provincial level. To the best of our knowledge, no Chinese city-level IO tables have incorporated any firm heterogeneity information. This paper is the first effort to fill this gap.

Several city-level MRIO tables have been developed for mainland China using either bottom-up or top-down methods. For instance, the city-level MRIO tables for Jing-Jin-Ji urban agglomeration are constructed based on 11 prefectural-city single region input-output (SRIO) tables of Hebei and those of Beijing and Tianjin^31,65^. Such a bottom-up method uses SRIO tables for different cities compiled from survey methods as the domestic transaction matrices. This approach allows one to reliably disaggregates city trade by sector to form inter-city transaction matrices. However, such an approach is very costly and often hampered by a lack of data because statistical agencies in most cities usually do not compile IO tables. As a result, the top-down approach, i.e., a non-survey mathematics-based proportional method, is widely used in inter-city IO table construction. It is often carried out through commodity balance (CB) or location quotient (LQ) methods. For example, due to the absence of updated survey-based city-level SRIO tables, the inter-city IO table for 11 prefecture-cities of Hebei for 2012 was updated using an entropy-based CB method^66^. Such a method is also used to create MRIO tables covering about 300 Chinese cities and four provinces (Qinghai, Yunnan, Hainan, and Tibet) for mainland China in the years 2012, 2015, and 2017, and is also applied to a 41-city table in the Yangtze River Delta city cluster^67^. The Industrial Ecology Virtual Laboratory (IELab) constructed another Jing-Jin-Ji inter-city table using Flegg’s LQ method^68^. It attempts to construct a flexible compilation methodology for subnational MRIO tables based on non-survey methods by splitting the national IO tables of China^30^. This approach is further used to create a provincial-city MRIO table for ten major cities in China and a GBA MRIO table for nine prefecture cities in the Pearl River Delta, linking with IO tables of Hong Kong and Macao extracted from the global MRIO database EORA^69^. The details of all published Chinese subnational MRIO databases at city levels are shown in Table 1. None of them covered the complete prefectural-level cities in mainland China and integrated Hong Kong, Macao, and Taiwan. To address this gap, this study attempts to reconcile all accessible micro-level data from various sources with city and provincial-level aggregate statistics to compile the first complete inter-city IO database for the Greater China area.

**Table 1:** The list of Chinese subnational MRIO databases at city levels.

Authors/Group	Types	Evaluation	Regions	Sectors	Years	Methods of table collection
China Emission Accounts and Datasets (CEADs)^66^	Commodity by commodity	Producers’ price	11 cities in Hebei province	42 sectors	2012	Download from https://www.ceads.net/data/input_output_tables/ or requesting tables from authors of the reference
China Emission Accounts and Datasets (CEADs)^31,65^	Commodity by commodity	Producers’ price	Beijing, Tianjin, and 11 cities in Hebei	42 sectors	2012	Download from https://www.ceads.net/data/input_output_tables/ or requesting tables from authors of the reference
China Emission Accounts and Datasets (CEADs)^67^	Commodity by commodity	Producers’ price	41 cities in the Yangtze River Delta city cluster	42 sectors	2012, 2015	Download from https://www.ceads.net/data/input_output_tables/ or requesting tables from authors of the reference
China Emission Accounts and Datasets (CEADs)^91^	Commodity by commodity	Producers’ price	309 Chinese cities and four provinces (Qinghai, Yunnan, Hainan, and Tibet)	42 sectors	2012, 2015, 2017	Download from https://www.ceads.net/data/input_output_tables/ or requesting tables from authors of the reference
Industrial Ecology Laboratory (IELab)^30^	Supply and use table	Basic price and purchasers’ price	14 cities for Beijing-Tianjin-Hebei Region	42 sectors	Annual years from 2007 to 2020	Requesting tables on demand at https://ielab.info (support@ielab.info) or from authors of the reference
Industrial Ecology Laboratory (IELab)^92^	Industry by industry	Basic price	10 major cities (Beijing, Tianjin, Shanghai, Nanjing, Hangzhou, Wuhan, Guangzhou, Shenzhen, Chongqing, Chengdu) and rest of 27 provinces	57 sectors	2015	Requesting tables on demand at https://ielab.info (support@ielab.info) or from authors of the reference
Industrial Ecology Laboratory (IELab) and Eora^69^	Industry by industry	Basic price	11 cities for the Guangdong-Hong Kong-Macao Greater Bay Area	26 sectors	2012	Requesting tables on demand at https://ielab.info (support@ielab.info) or from authors of the reference

Compared with previous work, our database introduces two methodological innovations for compiling inter-city IO tables. The first major methodological innovation is that the database adopts a combination of bottom-up and top-down methods to estimate the structure of the inter-city IO tables. A variety of microdata is used to estimate the economic structure of each covered city, including economic census, annual surveys of industrial firms (ASIF), urban and rural household surveys, and product level custom trade statistics. From the bottom-up perspective, these micro-level data are aggregated to obtain structural estimates at the sectoral level for each city. For every prefectural-level city, both economic census and ASIF are used to estimate industrial sector structure of gross output and value added, urban and rural household surveys provide the structure of household consumption expenditure by major categories in final demand, and detailed custom trade statistics inform the product structure of imports/exports. The database further combines the structure estimates from microdata with China’s provincial and city-level aggregate statistics as constraints to provide more precise structural shares for intermediate inputs of production, consumption of final demand, and trade flow at the city level. From the top-down perspective, the database regionalizes provincial IO tables into city IO tables with the bottom-up structure estimates constrained by GDP of cities to obtain estimates of the row and columns controls for the city IO tables. The use of such rich micro-level data significantly improve the accuracy and quality of the structural estimate of the constructed IO tables at the city level. Furthermore, each sector-city pair is further split by firm ownership using the data of firm’s registration types and realized capital structures from economic census and ASIF. The firm-level data are aggregated into the three major ownership groups and compiled to be consistent with provincial IO sector classifications. This also applies a bottom-up and top-down combination process to reduce the “aggregation error” come from the product-mix problem in the IO compilation process.

Overall, such a combined bottom-up and top-down compilation approach based on various micro-level and macro-level data, not only can help others to develop the inter-city IO tables with treatments of firm heterogeneity that meets the needs of their research, but also facilitates efficient workflows for the benchmark year of 2023. Starting from benchmark year 2023, the fifth China Economic Census and the eighth China Input-Output Survey have been integrated into one, and the advent of detailed and consistent data based on such a joint work can be easily utilized for the future city-level MRIO compilations based on our methodology, which focus on reconciling different sources of data at different levels.

The second key methodological innovation is that the database applies a three-tier data architecture to organize the compilation of the inter-city IO tables. The first tier is the raw data for further processing, which stores enormous micro and macro data from various sources. The second tier is the derived data sets for the inter-city IO table compilation, including processed data at the city level from the first tier to estimate both the sector and firm ownership structure for each prefectural city. Aggregated statistics such as provincial input-output tables and GDP estimated by production, income, and expenditure methods are used as constraints to reconcile these data sets derived from micro data. In this stage, three intermediate data products are generated, which can be applied to various research topics: (1) prefectural-city SRIO tables by firm ownership for each province (335 prefectural-city SRIOs and urban and suburb SRIOs of Chongqing, total of 337 SRIOs); (2) provincial-wide prefectural-city MRIOs by firm ownership for 27 provinces and Chongqing; (3) full city-level MRIOs for mainland China (total of 340 cities, more details can be found in Supplementary Table 1). The third tier is the final inter-city IO tables, i.e., the ownership-based inter-city IO tables, including the HMT region for the four benchmark years.

After introducing the main methodological innovations of our database. Now we describe the three important features of the constructed city-level IO tables: First, the database is one of the largest-scale inter-city IO tables in the world to date. It contains all above-prefectural cities in the Greater China area with 335 prefecture-level cities from 27 provinces, four municipalities directly under the Central Government (i.e., Beijing, Shanghai, Tianjin, and Chongqing with separate urban and suburb regions, subtotal of five regions in the table) in mainland China, and Hong Kong, Macao and Taiwan (HMT), a total of 343 regions. Furthermore, it is the first time that benchmark SRIOs for Macao have been constructed, and benchmark years and sector classifications for IO tables from Hong Kong and Taiwan have been harmonised with SRIO tables of mainland China. Second, the database is the first city-level IO table to incorporate firm ownership information. It distinguishes domestically-, HMT-, and foreign-owned firms for every city and sector for each of the four benchmark years. At China’s prefecture-city level, the constructed tables are based on the 42-sector classification consistent with the official input-output classifications issued by the National Bureau of Statistics of China (NBS). The heterogeneity in firm ownership for each sector at the city level is identified based on the information on registration types and utilized capital structures from firm-level microdata. This information is used for categorizing firms into three major ownership groups. Then key variables are aggregated by sector-city pair for each group, enabling to obtain key variables by sector and firm types for each city. Finally, this large-scale high-resolution database uses a consistent method to cover a very long time period, i.e., four benchmark years 2002, 2007, 2012, and 2017. Therefore, it can be used in a diverse range of socioeconomic and interdisciplinary issues in the Greater China area at the city level.

By incorporating the firm heterogeneity into the complete inter-city IO for four benchmark years, this dataset not only expands the opportunities for users to carry out new economic and environmental policy-related applications to re-estimations of various hot issues in current literature such as trade in value added across cities, carbon footprint accounting, and spatial distribution of production and employment in the Greater China area, but also provides a new data source for linking inter-city IO tables with global ICIO for domestic and global production network analysis. Beyond its application, the construction process of this database also provide insights for other large economies with significant variations of subnational regions like China to build their own inter-city IO tables with treatment of firm heterogeneities.

## Methods

The inter-city IO tables we constructed cover 340 cities in mainland China with 42 commodity sectors distinguishing domestically-, HMT-, and foreign-owned firms. Additionally, they integrate the IO tables from Hong Kong, Macao, and Taiwan with 42 sectors but do not contain firm ownership information.

The 42-sector classifications used in our database are slightly different in each benchmark year. The city IO sectors are consistent with classifications of provincial input-output tables in corresponding input-output survey years of 2002, 2007, 2012, and 2017. The sectoral classifications used by NBS in each survey year were aggregated from the *Chinese Industrial Classification of National Economic Activities* (CSIC), which was established in 1984 and revised several times to capture the change in industrial structure of Chinese economic development. The classifications of provincial input-output tables also changed based on the corresponding CSIC that revised in each survey year, that is, IO tables 2002, 2007, 2012, and 2017 using CSIC Rev. 1994, CSIC Rev. 2002, CSIC Rev. 2011, and CSIC Rev. 2017, respectively. To capture the change in the classification, sectoral classifications of the four benchmark years in our database vary with those of provincial input-output tables published by NBS. This approach keeps more detailed industry level information but may lead to some issues in comparing the IO tables in different years, we construct concordance files between classifications of four benchmark years to facilitate the reconciliation of those slightly different classifications.

The cities covered in the inter-city IO tables are determined by the data availability in our micro databases. The cities included both in the 2008 Economic Census and *China Custom Trade Statistics* (see Supplementary Table 1) are selected, whereas all existing Chinese city-level MRIO tables we discussed earlier choose cities based on whether data are available in statistical yearbooks. There are 344 unique prefectural-level 4-digit location codes from the 2008 Economic Census and 563 unique 4-digit location codes in *China Custom Trade Statistics* during 2007–2009. We reconcile the location codes from both sources and generate a list of 340 above prefectural-level cities in mainland China that are common to both datasets. Adding Hong Kong, Macao, and Taiwan, the total number of regions in our database is 343.

**Fig. 1:**
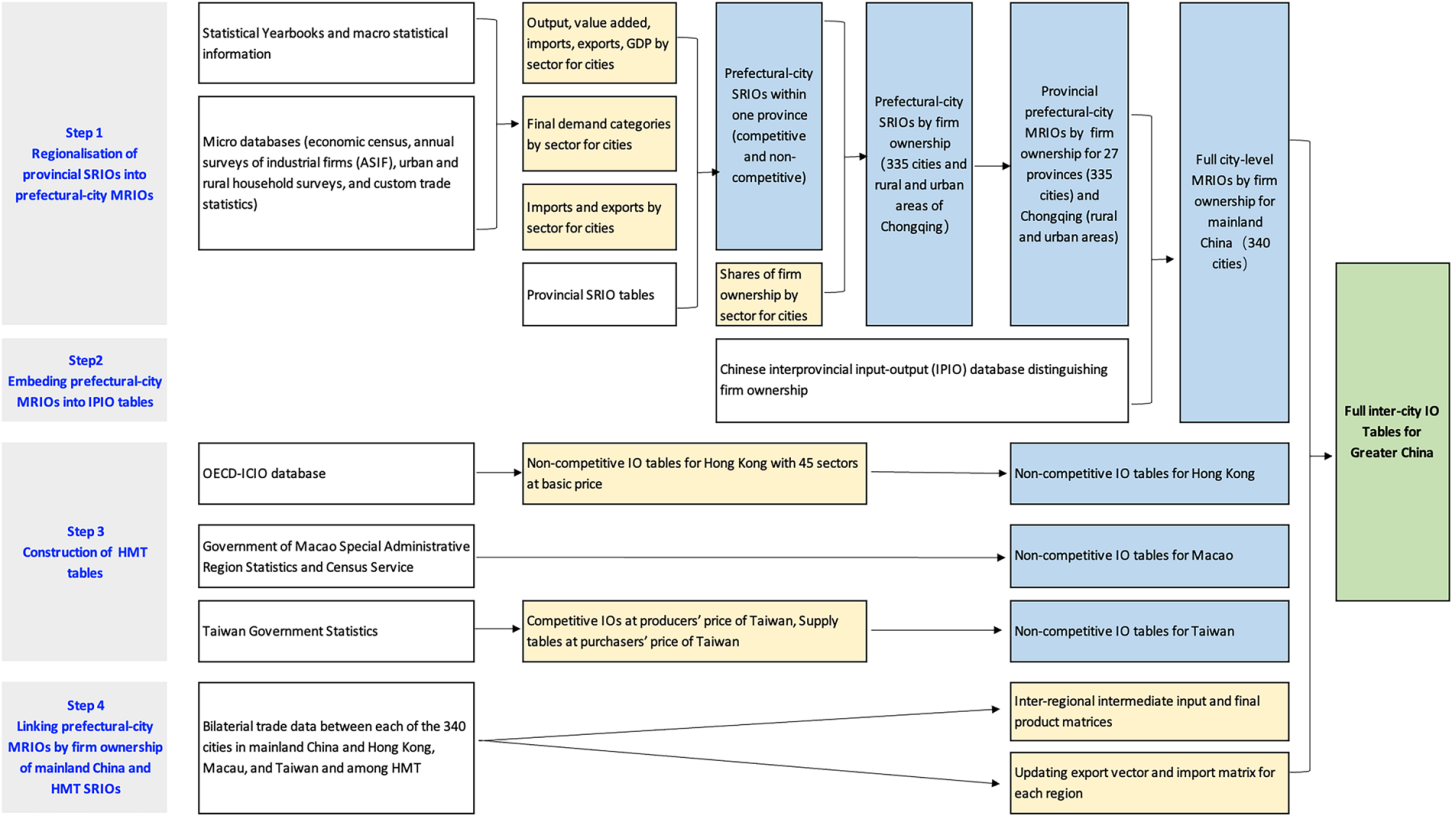
The schematic overview of the workflow for inter-city IO table construction. The grey section on the left outlines the four key steps in the compilation process. The white section denotes raw data sourced from various sources. The yellow section represents processed data at the city and sectoral levels, along with aggregated statistics. The blue section indicates derived datasets of intermediate input-output tables, and the green sections correspond to the final inter-city IO tables.

Figure 1 outlines our data reconciliation procedures into four main steps: (1) Regionalizing each provincial SRIO table into a prefectural-city MRIO table by three types of firm ownership for 27 provinces and Chongqing in mainland China. (2) Embedding the prefectural-city MRIOs into the interprovincial input-output (IPIO) tables to form a full city-level MRIO by firm ownership for mainland China. (3) Construction of non-competitive input-output tables with the consistent 42-commodity sectors for Hong Kong, Macao, and Taiwan, respectively. (4) Linking the full city-level MRIOs by firm ownership for mainland China with HMT non-competitive IO tables to form the inter-city IO tables for the Greater China area. The data in white boxes represent raw data from various sources, including macro and micro statistics and provincial input-output tables. The data in yellow boxes are the processed data by sector at the city level for further use, and the data in blue boxes show the various sets of derived (MR)IO tables constructed for this data project. The data in the green box is the final table described in this paper.

### Data sources and usages

Before introducing the detailed methodology, we document the sources of raw data used for deriving the necessary variables in the ownership-based inter-city IO table construction. These are categorized into three parts. The first part is the microdata, which allows us to estimate and aggregate the necessary variables for mainland China at the desired level (see Table 2).

**Table 2:** The list of raw micro-level data used in the ownership-based inter-city IO table construction.

Data	Involvement process	Sources
Firm-level output, value added, intermediate input, export delivery, registered type, registered capital, industry classification code, location code with 2004 for industrial sectors and 2008 for industrial and service sectors	Estimation of value added ratios by industrial industries for cities for construction of prefectural-city SRIO tables; Estimation of firm-ownership shares by sector for cities	Economic Census Database for 2004 and 2008
Firm level output, value added, intermediate input, export delivery, registered type, registered capital, industry classification code, location code, and employment for above-scale industrial firms for 2013 and 2015	Estimation of value added ratios by industrial industries for cities for construction of prefectural-city SRIO tables; Estimation of firm-ownership shares by sector for cities	Annual Survey of Industrial Firms (ASIF) from 1998 to 2015
Exports and imports for 2002, 2007, 2012, and 2017	Import supply and export demand estimates for 340 cities at 42 sectors	China Custom Trade Statistics
Household survey database for 2003, 2008, 2013, and 2018	Household consumption expenditure estimations in the final demand for 340 cities at 42 sectors	Chinese Household Income Project Survey, CHIP
Age structure of population at county-level of census years 2000, 2010, and 2020	Age structure of cities aggregated from counties using the close years between census years and the construction years	Tabulations on China Population Census by County for 2000, 2010, and 2020

Note: Detailed census data for 2004 and 2008 were provided by NBS, and they were not free for the public. *The National Economic Census Yearbooks for 2004 and 2008*^93,94^ provide more details about these two economic censuses conducted by China. The annual survey of industrial firms for 2013 and 2015 were provided by EPS China Data (permission required). The detailed customs data can be accessed by subscription through guidance on the official website of GACC^72^.

The second part is the data drawn from several different public available yearbooks (see Table 3). The data from these yearbooks are collected based on censuses and surveys organized by mainland China’s official statistical agencies.

**Table 3:** The list of data from yearbooks used in the ownership-based inter-city IO table construction.

Data	Involvement process	Sources
Sectoral output, Value added, Imports and exports of prefecture-level cities; GDP by primary, industrial, and tertiary industries of prefecture-level cities; Expenditure-side GDP of prefecture-level cities	Construction of prefectural-city SRIO tables within each province	City-level Statistical Yearbooks
Output, sales, value added, and employment of all sectors at city level for 2004, 2008, 2013, and 2018	Estimation of value added ratios by industrial, and tertiary industries for cities for construction of prefectural-city SRIO tables;Estimation of firm-ownership shares by sector for cities	Economic Census Yearbooks for nation and provinces for 2004, 2008, 2013, and 2018
Value added at city level	Estimation of firm-ownership shares by sector for cities, validating the value added estimation	China City Statistical Yearbooks
Expenditure-side GDP and its categories for nation, provinces, prefecture-level cities and counties for 2002, 2007, 2012, and 2017	Constraints for the final demand categories and table estimations	Various Statistical Yearbooks for nation, provinces, cities and counties
Population of cities	Estimation of household consumption expenditure by per capita of cities	Provincial Statistical Yearbooks

Note: Even though both city-level *Statistical Yearbooks* and *China City Statistical Yearbooks* report social and economic development at the city level, the data sources, and the statistics they covered are slightly different. The former is compiled by the local official statistics department, and each yearbook for a city only covers the information on this city. The latter is compiled by the Department of Urban Surveys, NBS, and it comprehensively covers the socio-economic data of most Chinese cities annually but with less detailed information on each city than each city’s statistical yearbook. Both are published by China Statistics Press, and cities’ statistic yearbooks can be found on the website of each local statistics department.

The last part includes the necessary regional and multiregional IO tables for compiling our the Greater China area inter-city IO tables. In addition to the regions of mainland China, it incorporates regional IO data for Hong Kong, Macao, and Taiwan, provided by the OECD and the corresponding governments (see Table 4).

**Table 4:** The list of IO tables and data for HMT used in the ownership-based inter-city IO table construction.

Data	Involvement process	Sources
Provincial SRIO tables for 2002,2007,2012, and 2017	Constraints in estimation of prefectural-city SRIO tables for each province; Estimation of value added ratios by tertiary industries for cities for construction of prefectural-city SRIO tables	China’s Reginal Input-Output Tables for 2002, 2007, 2012, and 2017
IPIO tables by three types of firm ownership for 2002, 2007, 2012, and 2017	Embedding prefectural-city MRIOs of each province into IPIO tables for forming full inter-city MRIOs for mainland China	Chen *et al*.^8^
Non-competitive IO tables for Hong Kong for 2002, 2007, 2012, and 2017 covering 45 sectors	IO table estimation for Hong Kong with 42 sectors	OECD-ICIO Database
National accounts and trade data, by total and 56-sector for 2002, 2007, 2012, and 2017	Constraints for Macao IO table and table construction	Government of Macao Special Administrative Region Statistics and Census Service
Competitive IO tables at producers’ price of Taiwan, Domestic and import transaction matrices for 2001, 2006, 2011 and 2016, covering 63 or 52 sectors	Convert IO tables into 42-sector tables	Taiwan Government Statistics
Supply tables at purchasers’ price of Taiwan 2002, 2007, 2012, and 2017 covering 50 sectors	Convert IO tables into 42-sector tables	Taiwan Government Statistics

### Step 1: Regionalizing provincial SRIO tables into prefectural-city MRIOs by firm ownership

This step focuses on the construction of prefectural-city MRIOs for 27 provinces and splitting Chongqing into urban and suburb regions, while the rest of the three municipalities directly under the Central Government of China (i.e., Beijing, Tianjin, and Shanghai) have been well-established in the IPIO tables (a total of 31 provincial-level regions in mainland China)^8^.

To regionalize a provincial SRIO table into a prefectural-city MRIO one by firm ownership, this step uses a revised methodology from Zheng, H. *et al*.^66^. Different from Zheng *et al*.’s work that estimated city SRIOs directly from a provincial SRIO table proportionally based on data from China’s city-level *Statistical Yearbooks*, our database combines microdata from multiple sources with macro data from Statistical yearbooks to estimate the sectoral structure of each city when constructing the city SRIOs within one province. In addition, firm ownership information from the Economic Census and ASIF at the city/sector level is used to form ownership-based city SRIOs. The description of these data sources is listed in Tables 2 and 3. The city SRIO tables distinguished by firm ownership are finally formed into MRIOs with estimated inter-city trade matrices for each province. Four sub-steps for the regionalization of one provincial SRIO table into prefectural-city MRIOs by firm ownership are as follows.

The preliminary prefectural-city SRIO tables compiled from multiple microdata and macro statistics often do not perfectly align, resulting inconsistencies. Thus, constrained balance methods are required to reconcile data conflicts in the tables. A bi-proportional balancing (RAS) method is used to resolve the multiple data sources of value added by sector and negative value of value added shares. When there is negative value of reported value added in certain sector due to government subsidy or profit loss in the initial SRIO tables, we apply the GRAS method instead of regular RAS^70^. GRAS method also used to fill in missing data in service sector when aggregated firm-level data into city-level by firm ownership because the two micro databases – Economic Census Database and ASIF do not cover firms in all 42 sectors and has to be supplemented by macro *Provincial Economic Census Yearbooks*. A maximum entropy model is applied to resolve the issues of incomplete information for cities when some data points in domestic output and input are missing or when a severe lack of information is confronted in the inter-city trade matrix.

#### Sub – step a: Estimating prefectural-city SRIOs within one province

**Fig. 2:**
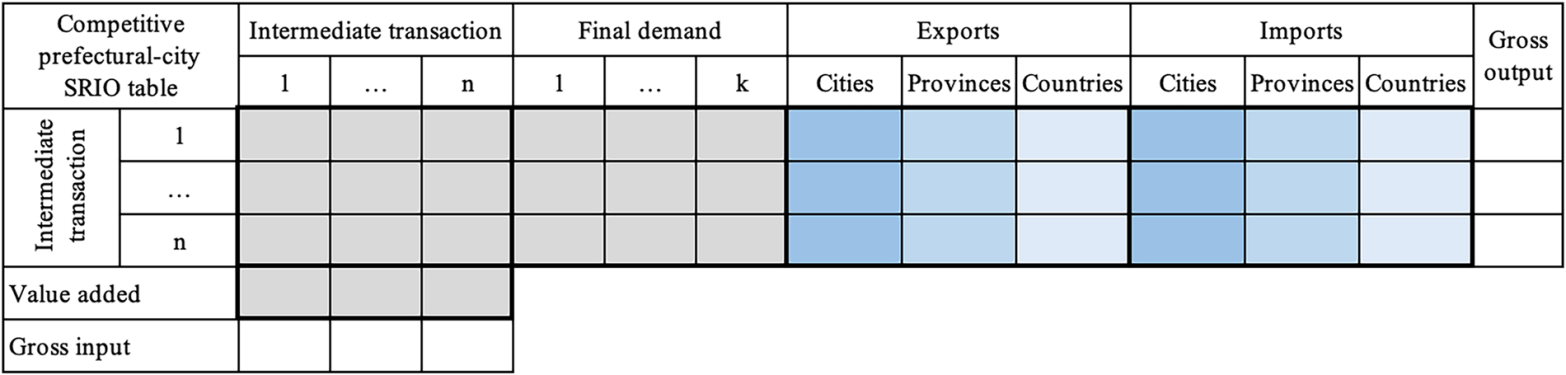
The structure of a competitive prefectural-city SRIO table. The grey areas represent the domestic economy of a city, encompassing intermediate transaction and final demand matrices, as well as value added vectors. The blue areas denote export and import matrices, and deep blue, medium blue, and pale blue correspond to one city’s exports to or imports from other cities within the same province, other provinces, and other countries, respectively.

To construct city-level SRIO tables within one province, as shown in Fig. 2, several matrices and vectors for all covered cities in the province need to be estimated. Elements in each cell of Fig. 2 represent different transactions for the city-level SRIO tables. For one city, these matrices and vectors include the domestic intermediate transaction matrices with commodities, domestic final demand matrices with categories, value added by sector, exports to other cities in the province, to other provinces, and to other countries by sector, respectively, imports from other cities in the province, from other provinces, and from other countries by sector, respectively. One city-level SRIO table must satisfy the basic balance constraint: gross output equals gross input for each sector. The gross output is the row sum of the intermediate output, final demand, and exports, while the gross input is the column sum of the intermediate input, value added, and imports. The provincial SRIO table constrains all the city-level SRIOs constructed in one province. The provincial SRIO table is available for each province, comprising 42 sectors, including five primary sectors, 21 industrial sectors, and 16 service sectors. The numbers and classifications of the sector and final demand categories of city SRIOs are consistent with those of the provincial one.

### Estimating gross output and value added by sector of cities

None of the data sources in Tables 2–4 includes gross output or value added by sector for all cities in our list. To address the issue of data availability without sacrificing spatial and sectoral heterogeneity, this paper combines multiple data sources to estimate city-sector level gross output and value added. The main data sources used include the micro-level Economic Census Database and ASIF, *Provincial Economic Census Yearbooks*, and city-level *Statistical Yearbooks*.

The original data on GDP by sector for each city is from city-level *Statistical Yearbooks*. The number of sector -level value added data available varies among cities and benchmark years. More than 50% of cities (175) in 2002 only provided value added data on agriculture and construction, while 60% of cities (203) in 2007 had such data over 20 sectors. Most cities in 2012 and 2017 provided less than 20 sector value added data, accounting for 49% (166) and 62% (210) of cities for 2012 and 2017, respectively. More details can be found in Supplementary Table 2. The gross output by sector for each city is estimated in several ways depending on data availability. The gross output of primary sectors for all cities can be directly taken from city-level *Statistical Yearbooks* or statistical bulletins. Apart from primary sectors, this study mainly relies on the data from census yearbooks to calculate the gross output by sector for each city. The *Provincial Economic Census Yearbooks* provide sector-level output or total sales information in secondary and tertiary sectors for each city within the province. As the economic census years do not align with the benchmark years of national and provincial IO tables, the gross output shares calculated from census years closest to these benchmark years were used as an approximation, i.e., the shares of 2004, 2008, 2013, and 2018 in Economic Census years were used for benchmark years 2002, 2007, 2012, and 2017, respectively.

Although combining statistics based on *Provincial Economic Census Yearbooks* and city-level *Statistical Yearbooks* provides sector-level gross output and value added data for most cities, there are still missing values for certain sectors in some cities. Moreover, merging two yearbook sources introduces inconsistencies that requires additional reconciliation steps. These issues are addressed by utilizing microdata from the detailed Economic Census Database and ASIF.

The detailed process of integrating data from *Provincial Economic Census Yearbooks*, city-level *Statistical Yearbooks* and microdata is as follows:

- First, we use the shares of gross output by sector for each city, calculated from microdata, to fill in the missing values in original shares of gross output from *Provincial Economic Census Yearbooks*, thus obtaining the complete shares of gross output by sector for each city in province they belong to. Here, we use ${{SY}}_{i}^{s}(k)$ to represent the shares of gross output by sector $i$ for city $s$ in province $k$. In this step, we also derive the shares of gross output by sector $i$ in city $s$ for a firm, and firms are indexed by ω. The output share by sector in a city for firm ω can be represented as ${{Sy}}_{i}^{s}(\omega )$. Therefore, the shares of gross output for a firm in the province are calculated as ${{SY}}_{i}^{s}(k){{Sy}}_{i}^{s}(\omega )$.
- Second, we use gross output and value added (the sum of compensation of employees, consumption of fixed capital, taxes less subsidies on production, and operating surplus) from the Economic Census Database and ASIF to calculate the ratio of value added to gross output at the firm level, denoted by ${r}_{i}^{s}\left(\omega \right).$ In the next several steps, we will aggregate these firm-level ratios into the city level by sector.
- Third, we multiply the shares of gross output by sector for each city within the province obtained in the first step by the benchmark gross output from the IPIO tables to obtain the benchmark gross output by sector for each city.
  1 $${Y}_{i}^{s}\left({\rm{k}}\right)={Y}_{k}^{s}\,{{SY}}_{i}^{s}\left(k\right)$$
  Here, ${Y}_{i}^{s}\left({\rm{k}}\right)$ represents the benchmark gross output by sector $i$ for city $s$ in province *k*. ${Y}_{k}^{s}$ is the benchmark output by sector $i$ in province $k$, which is from our benchmark IPIO tables. Subsequently, we first multiply ${{Sy}}_{i}^{s}(\omega )$ to get each firm’s benchmark gross output, then we further multiply this by the value added ratio calculated in the second step to estimate the benchmark value added by sector in a city for each firm. At the end of this step, we aggregate the ${\varOmega }_{i}^{s}$ firms in the $(i,s)$ city/sector pair of a province to get the benchmark value added by sector for each city (see the following Eq. 2):
  2 $${V}_{i}^{s}\left({\rm{k}}\right)=\mathop{\sum }\limits_{\omega =1}^{{\varOmega }_{i}^{s}}{{[Y}_{k}^{s}{SY}}_{i}^{s}(k){{Sy}}_{i}^{s}(\omega )]{r}_{i}^{s}(\omega )$$
- Fourth, after calculating the benchmark value added by sector for each city, the aggregated the ratio of value added to gross output for each city/sector pair $(i,s)$ can be obtained as:
  3 $${r}_{i}^{s}\left({\rm{k}}\right)=\frac{{V}_{i}^{s}\left({\rm{k}}\right)}{{Y}_{i}^{s}\left({\rm{k}}\right)}$$
- Fifth, we categorise the cities into two groups for each sector in the province according to whether they report sector-level value added in city-level *Statistical Yearbooks*. One group reports actual value added data, and the set of city indices in this group is denoted as ${G}_{r}$. The other group, which does not report actual value added, is denoted as ${G}_{{nr}}$. We then calculate the weights of the two groups for each sector using the estimated value added obtained in the previous step:
  4 $${W}_{r}=\frac{\sum _{i\in {G}_{r}}{V}_{i}^{s}\left({\rm{k}}\right)}{\sum _{i\in {G}_{r}}{V}_{i}^{s}\left({\rm{k}}\right)+\sum _{i\in {G}_{{nr}}}{V}_{i}^{s}\left({\rm{k}}\right)}$$
  5 $${W}_{{nr}}=\frac{\sum _{i\in {G}_{{nr}}}{V}_{i}^{s}\left({\rm{k}}\right)}{\sum _{i\in {G}_{r}}{V}_{i}^{s}\left({\rm{k}}\right)+\sum _{i\in {G}_{{nr}}}{V}_{i}^{s}\left({\rm{k}}\right)}$$
- Then, for the group of cities that reports actual value added in the statistical yearbooks, we calculate their shares of value added by sector within the group using actual data.

6 $${{{Sv}}_{i}^{s}(k)}_{r}=\frac{{{V}_{i}^{s}\left({\rm{k}}\right)}^{\ast }}{\sum _{j\in {G}_{r}}{{V}_{j}^{s}\left({\rm{k}}\right)}^{\ast }}$$

Here, the data from official census yearbooks or statistical yearbooks is labelled with superscript “*”. For cities that do not report actual value added, we use the estimated value added by sector obtained in the third step to calculate their shares of value added within the group.

7 $${{{Sv}}_{i}^{s}(k)}_{{nr}}=\frac{{V}_{i}^{s}\left({\rm{k}}\right)}{\sum _{j\in {G}_{{nr}}}{V}_{j}^{s}\left({\rm{k}}\right)}$$

Once we get the shares of the cities within their respective groups, we multiply them by the weights of their groups for each sector to obtain the shares of value added by sector of each city within the province.

8 $${{Sv}}_{i}^{s}(k)=\{\begin{array}{c}{{{W}_{r}{Sv}}_{i}^{s}(k)}_{r},i\in {G}_{r}\,\\ {W}_{{nr}}{{{Sv}}_{i}^{s}(k)}_{{nr}},i\in {G}_{{nr}}\end{array}$$

- Next, we use the GRAS method to adjust the ${{Sv}}_{i}^{s}\left(k\right)$ based on the constraints generated by provincial-level value added by sector and the GDP for each city reported by city-level *Statistical Yearbooks*.
- Finally, we use the shares of value added from the previous step and the value added ratio from the fourth step (see Eq. 3) to deduce the proportion of gross output.

Once all the shares of gross output and value added by sector for each city within its province are ready, we calculate gross output and value added by sector for each city by multiplying the benchmark values of each province from the IPIO tables by the calibrated shares.

The principle of the above steps is to use the variables of the IPIO tables as general constraints, to use data from official reports as a basis for splitting as much as possible, and to supplement it with microdata in cases where official data of suitable accuracy are not available. Based on the procedures outlined above, we obtain gross output and value added by sector of each city using a consistent approach and maximising the utilisation of available information. The estimates are consistent with the IPIO tables at sectoral aggregates, and the sector-level value added ratios for each city are controlled by microdata, and the relative sizes of sector-level value added for each city are derived from actual data. Here, we use actual value added from city-level *Statistical Yearbooks* as the main target for calibration since the data on value added are more reliable than the data on gross output at the city/sector level. Combining city-level data from city-level* Statistical Yearbooks* with microdata-based value added ratios and shares of gross output, the estimation of gross output and value added by sector is more robust over the benchmark years than estimations that only used the value added by more aggregated sectors for primary, secondary, and tertiary industries. In addition, controlling the sectoral structure of each city using microdata can partially mitigate the over-reporting issue caused by local governments, which is common in the GDP data reported by local governments in mainland China^71^.

Note that when calculating the value added ratio using microdata, some results are unreasonably large or even greater than one, which is theoretically not allowed. To mitigate the inconsistent issues caused by measurement errors, we impose restrictions on value added ratios by assuming that the difference in value added ratios across cities within one province is less than the largest difference in value added ratios across provinces (more details about processing microdata can be found in Sub-step b). This assumption implies greater heterogeneity in production at the provincial level than across cities within each province. We use a regression interpolation method to replace all outliers (value added ratio greater than 1) in the original estimates. In case the city-sector level value added ratio is still missing after all these steps, we use the provincial-level value added ratio at the sector level to fill in the missing data.

### Estimating components of domestic output and domestic input of cities

The gross output by sector of each city can be further divided into domestic output and exports to other countries. The gross input by sector can be divided into domestic input and imports from other countries. Exports to other countries by sector and imports from other countries by sector are aggregated from 8-digit HS product level *China Custom Trade Statistics* for the years 2002, 2007, 2012, and 2017, which are provided by the General Administration of Customs of the People’s Republic of China (GACC)^72^. The domestic output by sector of cities equals gross output minus exports to other countries by sector. The domestic input by sector of cities is estimated by assuming the ratio of intermediate input to domestic total input is identical between a city and its province. The intermediate input by sector of cities is calculated by using the provincial input structure multiplied by gross output by sector of cities. The domestic input by sector of cities is obtained by total intermediate input by sector of cities multiplying the provincial ratio of imported intermediate input to total input, with the constraint that the sum of estimated cities’ domestic input should equal the total domestic input by sector of the province.

Domestic output is further divided into local output by the cities itself, exports to other cities in the province, and exports to other provinces. Correspondingly, domestic input is separated into input from local firms in these cities, imports from other cities in the province, and imports from other provinces. The six components of domestic output and domestic input are estimated by the maximum entropy model^66,73–75^, which is constrained by balance conditions of the city’s output and input, by provincial domestic exports/imports in provincial SRIOs, and by the probability of the city’s output and input in the maximum entropy model, respectively.

### Estimating intermediate transaction matrices of cities

Output to and input from local are intra-city matrices of cities, including intermediate transaction matrices, final demand matrices, and value added vectors. The value added vector of each city has been computed from a combination of microdata and statistics in the statistical yearbooks as discussed earlier, and thus the estimations of intermediate transaction matrices and final demand matrices of cities are key tasks.

The intermediate transaction matrix of each city in the province uses the provincial technical coefficient matrix from their provincial SRIO table as the proxy multiplied by the sectoral output of each city to obtain the initial intermediate transaction matrix for each city, the same method as used in the Zheng *et al*.^66^, which is assuming that provincial technical coefficient matrix reflects the average technical coefficients for all cities in the province. The provincial SRIOs are taken from *Chinese Regional Input-Output* Tables^76–79^. The column sum of each sector of the intermediate transaction matrix should equal the difference between input from the city locally and value added. The row sum of each sector of the intermediate transaction matrix should equal the difference between the local output of the city and its final demand.

### Estimating final demand matrices of cities

The final demand matrix of each city for consuming domestically produced 42 commodities comprises five categories: urban household consumption expenditure, rural household consumption expenditure, government consumption expenditure, gross fixed capital formation, and changes in inventories. The final demand matrix of each city by sector and by category is estimated from various city-level micro and macro data sources rather than what did by Zheng *et al*.^66^, where the structure of final demand from the provincial SRIOs is uniformly used to distribute each city’s total final demand to the 42 commodities in the five categories.

Both urban and rural household consumption expenditures by sector are estimated by consumption category and then disaggregated into 42 commodity sectors. Complete household consumption expenditure by major consumption categories is obtained from city-level *Statistical Yearbooks, Household Survey Yearbooks*, and *CHIP*. The number of cities covered in the three data sources differs between urban and rural areas and varies over the four benchmark years. Less than 10% of cities (30, 26, and 25 in 2007, 2013, and 2018, respectively) in city-level *Statistical Yearbooks* provide household consumption expenditure for only three years 2007, 2013, and 2018. Most cities are covered in *Household Survey Yearbooks* and *CHIP*, these data also differ slightly by urban and rural areas and by time. For cities that appear in both data sources, we give priority to the official data source, i.e., *Household Survey Yearbooks*. See Table 5 for details.

**Table 5:** A summary of the numbers of cities covered in three data sources for estimating household consumption expenditure. ‘—’ indicates no data.

Year	City-level Statistical Yearbooks		Household Survey Yearbooks		Chinese Household Income Project (CHIP)	
	Urban	Rural	Urban	Rural	Urban	Rural
2002	—	—	152	139	61	107
2007	30	30	236	206	98	82
2013	26	26	160	143	123	121
2018	25	25	223	214	154	125

Household consumption expenditure by consumption category is obtained in several ways depending on the availability of data from three sources. The detailed process of integrating city-level *Statistical Yearbooks* and *Household Survey Yearbooks* with micro survey database *CHIP* is as follows:

- Household consumption expenditure of eight major consumption categories can be directly extracted from cities’ expenditure-side GDP in city-level *Statistical Yearbooks, but* this data source is only available in a small proportion of the list of cities in our database (no more than 30 shown in Table 5).
- We use household expenditure per capita to estimate household consumption expenditure. All three data sources can provide household expenditure per capita of eight major consumption categories^80^. Because the micro survey data for each household in the CHIP database only cover 2002, 2007, 2013, and 2018, of which the latter two years are inconsistent with the four benchmark years in our database. We deflate Household expenditure per capita in three data sources for 2013 and 2018 to benchmark years 2012 and 2017 by cities’ consumer price indices and the GDP growth rate of corresponding years. Household expenditure per capita is then converted to household consumption expenditure of each city by multiplying the population of the city.
- A small part of cities in our database city list still lacks household expenditure and/or household expenditure per capita by consumption category. The total household expenditure of such cities from city-level *Statistical Yearbooks* is disaggregated by the consumption structure of a similar city to obtain the household expenditure by consumption category of the cities. The choice of a similar city is based on the age structure of the cities’ population, which disaggregates18 age groups into three major groups, including age groups of 0–19, 20–59, and 60 above. We use the largest population group of 20–59 as the representative combining with GDP per capita of cites to select the similar cities.
- No similar city could be found for a few cities, shares of total retail sales of consumer goods of a city in the province total are used to split provincial household consumption expenditure in the provincial SRIOs to obtain the household expenditure by consumption category of such cities.

When household expenditure by consumption category for all 340 cities is ready, the eight major consumption categories are reconciled into the 42 commodity sectors using concordance matrices for transformation constructed by a binary classification matrix with sectoral employment of cities as proxies^30^. The sum of urban and rural household consumption expenditures by sector of cities in the province is constrained by household consumption expenditures from cities’ expenditure-side GDP. The rest of the three categories of final demand: government consumption expenditure, gross fixed capital formation, and changes in inventories – are estimated by splitting corresponding components of cities’ expenditure-side GDP into 42 commodity sectors based on the sectoral structure of the provincial SRIO table, which assumes that sectoral structures of government consumption, gross fixed capital formation, and changes in inventories of each city are identical with those of the province. When components of the expenditure-side GDP of some cities are unavailable, general government expenditure and total investment in fixed assets are used as proxies for government consumption expenditure, gross fixed capital formation and changes in inventories, respectively, to divide the rest of the provincial totals and then decompose them into sectors via final demand structures of the provincial SRIO table. The sum of government consumption expenditure by sector, gross fixed capital formation by sector, and changes in inventories by sector of each city in the province are constrained by the corresponding category of final demand by sector in the provincial SRIO table, respectively.

### Adjusting initial estimates to meet constraints of city SRIOs

The initial estimates of intermediate transaction and final demand matrices are further adjusted using the cross-entropy model to satisfy the constraints of each city SRIO table in the province. Three basic constraints must be satisfied: the balance constraint that the row sum of each sector is equal to the column sum of the corresponding sector for each city SRIO table, the column constraint of sectoral intermediate transactions is equal to the difference between gross input and value added by sector, and the row constraint of sectoral intermediate transaction plus final demand is equal to the difference between total output and net export (exports minus imports)^81,82^ by sector. The entropy between the objective and the initial distributions can be minimized and modified to the initial distribution to meet the constraints^83^ to generate new matrices of intermediate transactions and final demand.

Up to now, matrix and vector estimates for all the components of competitive prefectural-city SRIOs in each province have been constructed, as shown in Fig. 2.

### Converting city SRIOs from competitive- to non-competitive-type

**Fig. 3:**
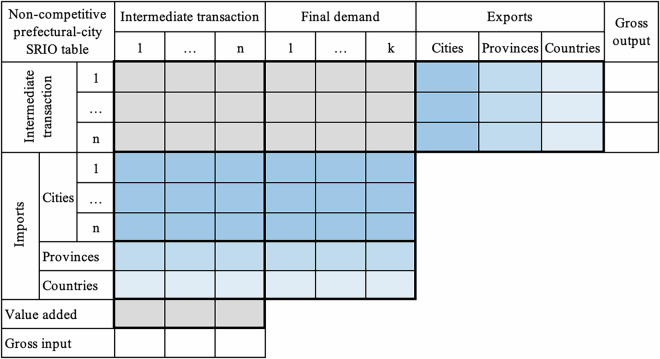
The structure of a non-competitive prefectural-city SRIO table. The colour meanings remain consistent with Fig. 2. The key difference is that imports of one city from other cities within the same province (deep blue), other provinces (medium blue), and other countries (pale blue) are distinguished into intermediates transaction and final demand rather than being represented as total import vectors in Fig. 2.

The provincial SRIOs published by NBS of China are competitive-type IO tables, where matrices of intermediate transaction and final demand include imports. The city SRIOs regionalised from provincial SRIOs are thus competitive-type ones without distinguishing imports from the intermediate transaction and final demand matrix, which cannot be used directly to further estimate domestic and inter-city trade matrices for prefectural-city MRIO. The conversion of the competitive city-level SRIOs into non-competitive tables is carried out by assuming a fixed proportion of imports and inflows in intermediate and final demand matrices^11,66^ to split the above competitive-type ones into domestic and import matrices for intermediate transaction and final demand, respectively. The conversion for each city yields an SRIO table for domestic commodities and imports, distinguishing intermediate transactions and final demand. The import vectors of each competitive-type city SRIO in the conversion are divided into 42 commodity matrices of imports from other cities in the province, other provinces, and other countries, respectively. The matrices for provincial and other countries’ imports aggregated by column, together with matrices for other cities in the province, are located at the bottom of the intermediated transaction and final demand matrix in each non-competitive city SRIO, as shown in Fig. 3.

At this step, the non-competitive city SRIOs regionalized within one province for all 27 provinces (a total of 335 prefectural-city SRIOs) and Chongqing (two SRIOs for urban and suburb areas, respectively) are constructed (a total of 337 prefectural-city SRIOs) for further split by information of three types of firm ownership.

#### Sub – step b: Disaggregating prefectural-city SRIOs by firm ownership

**Fig. 4:**
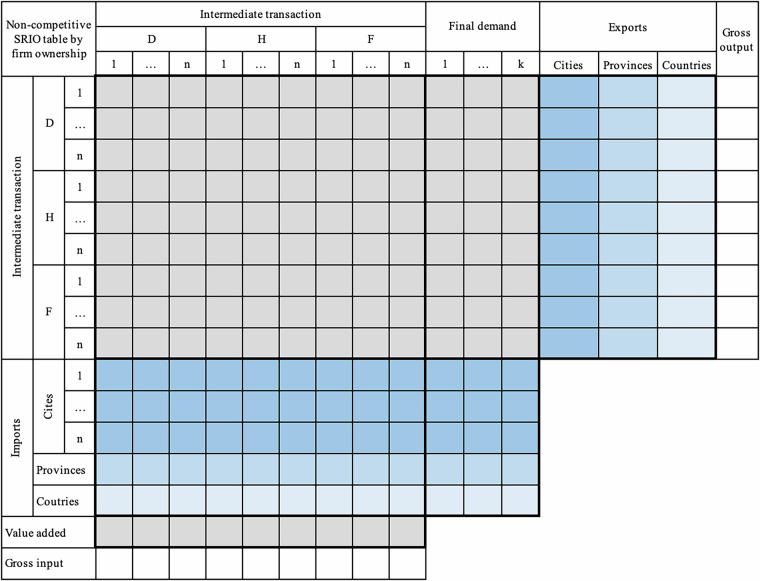
The structure of a non-competitive prefectural-city SRIO table by firm ownership. The colour meanings remain consistent with Figs. 2 and 3. The key distinction is that each sector in Fig. 4 is further classified by ownership, distinguishing domestically-, HMT-, and foreign-owned firms.

This step aims to disaggregate sectors in non-competitive prefectural-city SRIOs by three types of firm ownership (Fig. 4). The disaggregation is carried out for each prefectural city in all 27 provinces and the urban and suburban areas of Chongqing in mainland China. In this step, we obtain the first set of data products in our database – prefectural-city SRIOs by firm ownership (a total of 335 SRIOs in all 27 provinces and two in Chongqing), depicting economic relationships with firm heterogeneity for a single prefectural city.

### Data sources for estimating shares of key variables by firm ownership

The sectoral shares by firm ownership of gross output, value added, intermediate input, and export delivery at the city level are estimated mainly by two firm-level data sources, the Economic Census Database and ASIF. Similar to the approach for estimating city-sector level gross output and value added in the previous sub-step, the detailed Economic Census for 2004 and 2008 and ASIF for 2013 and 2015 were chosen as the closest year of estimated firm-ownership shares as the proxies corresponding to the benchmark years, i.e., the shares of 2004, 2008, 2013, and 2015 were used for the benchmark years of 2002, 2007, 2012, and 2017, respectively. As the two micro databases don’t contain firms in all the 42-sector for all the prefectural-city SRIOs (the number of firms covered can be found in Table 6), we first fill in the missing data using a GRAS method and *Provincial Economic Census Yearbook*s for 2004, 2008, 2013, and 2018 as supplementary data sources for the estimation. *Provincial Economic Census Yearbooks* report the output or sales by firm ownership for a number of sectors. If there are still some missing variables (most of these cases happened in service sectors), we use the GRAS method, which is similar to what we used in **sub-step a** to fill them in. That is, given the estimated shares of the year 2008, from which detailed micro data on service are available, we update the new set of shares at city-sector level by firm ownership. This update is carried out by controlling the city-level output/sales/value added by sector and the aggregated-city level statistics by firm ownership in their cities.

**Table 6:** A summary of the numbers of firms covered in the micro database, Economic Census Database for 2004 and 2008 and ASIF for 2013 and 2015. It is the same as Table 3 and Table 4 in the Chen *et al*., 2023.

Year	Number of observations	Description
2004	1,375,148	All active firms in industrial sectors only
2008	5,228,724	All active firms in all sectors except the primary sector
2013	344,875	All above-scale firms in industrial sectors only
2015	305,498	All above-scale firms in industrial sectors only

### Ownership classification of firms in the micro databases

The ownership type of a firm is tagged by the registered type of the firm (‘*qiye dengji zhuce leixing*’ in Chinese) in the Economic Census Database or ASIF, which has been defined as 25 types based on firms’ registered capital by the NBS of China. The 25 types of firms are further classified into three major groups, i.e., domestically owned, Hong Kong, Macao, and Taiwan-owned (HMT-owned), and foreign-owned (see Table 7 for detailed classification). Domestically owned firms are identified using the type of registration information. In addition to the wholly HMT and wholly foreign investment enterprises, joint-venture firms are classified as either HMT- or foreign-owned if at least 25% of their utilized capital was HMT- or foreign-owned, respectively. Utilized capital shares are only used for classifying joint-venture firms because the type of firm registration, and shares of utilized capital are inconsistent for some firm level observations.

**Table 7:** Registration information of firms.

Registered ID	Registered type	Ownership in the paper
110	State-owned enterprises	domestically owned
120	Collective enterprises	domestically owned
130	Joint-stock enterprises	domestically owned
141	State-owned associated enterprises	domestically owned
142	Collective associated enterprises	domestically owned
143	State-owned and collective associated enterprises	domestically owned
149	Other associated enterprises	domestically owned
151	Wholly state-owned company	domestically owned
159	Other limited liability company	domestically owned
160	Incorporation	domestically owned
171	Wholly private enterprises	domestically owned
172	Private partnership	domestically owned
173	Private limited liability company	domestically owned
174	Private Incorporation	domestically owned
190	Other domestic enterprises	domestically owned
210	Equity Joint venture enterprises (HMT)	HMT-owned
220	Contractual joint venture enterprises (HMT)	HMT-owned
230	Wholly HMT investment enterprises	HMT-owned
240	HMT Investment limited liability company	HMT-owned
290	Other HMT investment enterprises	HMT-owned
310	Sino-foreign investment equity joint venture enterprises	foreign-owned
320	Sino-foreign investment contractual joint venture enterprises	foreign-owned
330	Wholly foreign investment enterprises	foreign-owned
340	Foreign investment limited liability company	foreign-owned
390	Other foreign investment enterprises	foreign-owned

Note: NBS of China uses a 3-digit code to classify registered types of firms. The firms with registered ID commenced with “1” are classified as domestically owned. The firms with registered type ID starting with “2” are classified as “HMT.” The rest of the firms are treated as foreign-owned firms.

### Estimating sectoral shares of key variables by firm ownership at the city level from micro databases

When all firms in the Economic Census Database and ASIF are classified into one of the three ownership types, four key variables (i.e., gross output, value added, export delivery value, and intermediate input) are aggregated by city and by 42-IO sector, respectively. Then shares of the variables by firm ownership are calculated. Most provinces do not report values of sales or output in primary and tertiary sectors at the city level, but the number of employments, and thus shares of sectoral employment at the city level, are used as a proxy for estimating sales and output in primary and tertiary sectors. The primary, postal service, and government organisation sectors are domestically dominated in China, and there is no obvious heterogeneity in firm types across cities for these sectors. We thus assume the same shares by firm types within each province. For sectors that only report gross output or sales, shares of those four key variables are approximated using the shares of gross output or sales.

### Special treatments of key variables from micro databases

(1) Negative values exist in key variables that contradict theoretical values, which is only less than 0.01% of observations in the micro databases. Negative output, negative employment, and negative utilized capital in the micro databases were assumed as the results of measurement errors and directly omitted from our calculations. (2) Measurement error can significantly hamper the accuracy of the results when only very few firms are sampled in a specific sector of a city. A severe issue caused by this problem is that the value added ratios of some cities are greater than one. To tackle this issue and mitigate the inconsistency caused by the measurement error, sectoral or city-level statistics reported by *Economic Census Yearbooks* are used as benchmark results to calibrate the shares of each city in a province (i.e., the shares of firms’ total output or value added in a city equal to the ones from *Economic Census Yearbooks*). (3) The industrial classification of firms in micro databases is based on China’s standard industry classification (CSIC); they are mapped into IO sector classifications for each benchmark year using concordance matrices of the two industrial classifications. (4) The value added by firms was not reported for all years. Value added for the missing years are calculated by production-side approach – output minus intermediate input and value added tax payable or income-side approach – the sum of depreciation, labour compensation, net tax of production, and operating surplus. (5) Around 30% of firms in the 2008 Economic Census did not report their registered types, and thus, the types of these firms were identified by comparing their HMT- and foreign-owned shares of utilised capital with the 25% threshold. (6) The estimation of ownership variables is primarily based on micro-data when available, as it captures greater heterogeneity across cities within each province compared to statistical yearbook reports. In the case of municipalities, abnormal values are manually adjusted based on census yearbooks or city-level *Statistical Yearbooks* due to their richer industry-city level data reported in yearbooks compared to other prefectural cities.

When sectoral shares of key variables by firm ownership at the city level are estimated, they are used to disaggregate the above non-competitive city-level SRIOs for all 27 provinces and Chongqing in Sub-step a to obtain 337 prefectural-city SRIOs distinguishing three types of firm ownership (Fig. 4) for further use in Sub-step c.

## Sub – step c: Estimating inter-city trade flows by firm ownership in one province

### Estimating inter-city trade flows by sector between cities in one province

One of the major issues in constructing inter-city IO tables is a severe lack of inter-city trade data, and statistical information about exports to or imports from other cities is rare. Therefore, the maximum entropy doubly constrained gravity model is thus used to estimate inter-city trade flows by sector, as used in Zheng *et al*. The approach assumes that inter-city trade by sector is proportional to inter-city total exports and total imports and anti-proportional to transport costs^75^. For each city, both the exports by sector to other cities in the province and the imports by sector from other cities in the province estimated in Sub-step a are taken as column-sum and row-sum constraints, respectively. The transport costs by sector are estimated by using the straight-line distance extracted from the Geographic Information System (GIS) for a shipment of the commodity sector between cities. The estimated inter-city trade flows for each sector are constrained by local intermediate and final demand transactions in the province, which is the difference between the total intermediate and final demand transactions and the inflow and outflow transactions in the IPIO tables. The transport costs are only applied to shippable commodities, while transport costs for non-shippable commodities such as services are assumed to be zero.

### Disaggregating sectors of inter-city trade flows by firm ownership

Using the sectoral shares of gross output by firm ownership for each city in the province estimated in Sub-step b to disaggregate inter-city trade matrices in the province, we obtain inter-city trade matrices by firm ownership.

## Sub – step d: Compiling a full prefectural-city MRIO table within one province

**Fig. 5:**
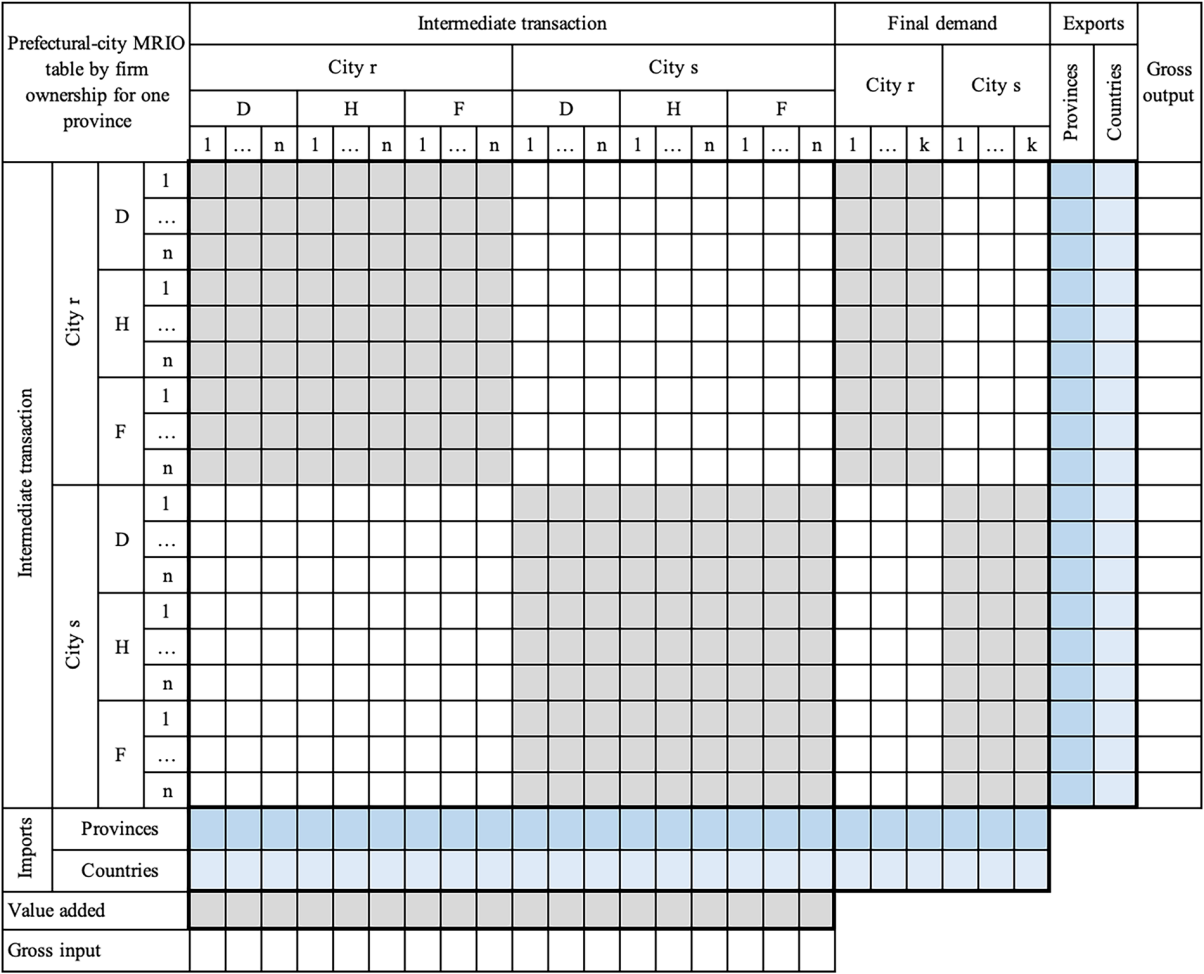
The structure of a prefectural-city MRIO table by firm ownership for one province. The colour meanings remain consistent with Figs. 2, 3, and 4. The key distinction is that in this figure, imports from other cities within the same province – previously represented in deep blue – are now shown in grey. This change reflects the treatment of cities as equal economic regions in the provincial MRIO table, capturing both domestic and inter-city transactions along with final demand.

A full prefectural-city MRIO table by firm ownership in one province is formed, as shown in Fig. 5. The diagonal intermediate and final demand matrices of the prefectural-city MRIO table by firm ownership in one province are available in the non-competitive prefectural-city SRIOs by firm ownership constructed in Sub-step b. Combining prefectural-city SRIOs by firm ownership with inter-city trade matrices by firm ownership, the off-diagonal trade matrices between cities in Fig. 5 can be constructed. The combination is carried out by calculating the inflow purchase coefficients (IPC) matrix for each sector based on inter-city trade matrices, where IPC for a commodity sector is the proportion of all inter-city input for the sector provided by each city. The off-diagonal trade matrices in the prefectural-city MRIO table are estimated using the imported intermediate and final demand matrices by firm ownership multiplied by IPC, respectively^84,85^.

The complete prefectural-city MRIO table is then constructed with sectoral output, value added, exports to other provinces, exports to other countries, imports from other provinces, and imports from other countries by firm ownerships from non-competitive prefectural-city SRIOs by firm ownership and balanced by row sum equalling column sum. The provincial SRIO table constrains each step of the compilation. After this step, a full prefectural-level MRIO table by firm ownership for one province is finally constructed.

The above step-by-step approach provides a general framework for building a prefectural-city MRIO table by firm ownership for one province. Except for three municipalities directly under the Central Government (Beijing, Tianjin, and Shanghai) that have been constructed in the IPIO tables, 27 prefectural city-level MRIOs by firm ownership within their provincial boundary and urban and rural regions of Chongqing were constructed, respectively. In this stage, we obtain the second set of intermediate data products, i.e., provincial-wide prefectural-city MRIOs by firm ownership for 27 provinces and Chongqing with two regions, depicting the local economic geography for each province.

### Step 2: Embedding provincial prefectural-city MRIOs into IPIO tables to form full prefectural-city MRIOs by firm ownership for mainland China

The methodology for nesting provincial city-level MRIOs to create a full city-level table for all provinces has been constructed by constraining city-province trade using the provincial MRIO table^31,54,84^. Specifically, an innovative and consistent Chinese IPIO database distinguishing firm ownership is used as a constraint when constructing the full prefectural-city MRIOs by firm ownership. Because both provincial prefectural-city MRIOs and IPIO tables are distinguished by three types of firm ownership, we will not mention the ownership to simplify our description.

**Fig. 6:**
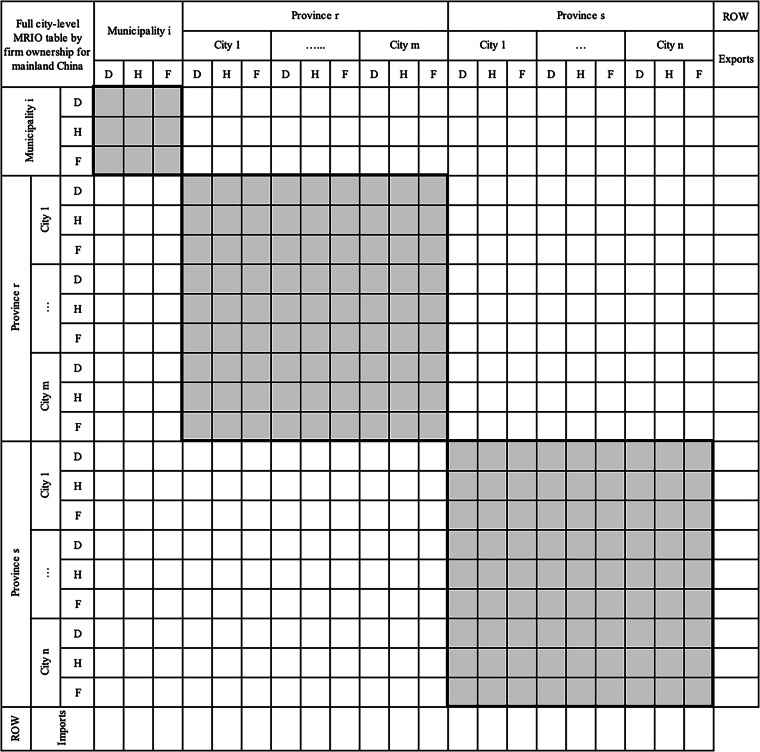
The structure of a prefectural-city MRIO table by firm ownership within the IPIO table. Provincial prefectural-city MRIO tables for each municipality and province are represented in grey, illustrating the embedding process and structure of these MRIO tables within the IPIO framework.

The illustrated structure of the full prefectural-city MRIOs for mainland China is shown in Fig. 6. For simplicity, it only shows one municipality and two provinces as an illustration and doesn’t detail the sectors in each region. In this step, Beijing, Tianjin, and Shanghai, the three municipalities directly under the central government, are provincial-level regions in China, are regarded as city-level regions in our database and thus remain unchanged as is in the IPIO table. Although Chongqing is also a municipality, we divide it into urban and rural areas because its rural area and population are very large which is suitable to treat them as one separate region from the urban area. We only insert the two-region table of Chongqing and the rest of the 27 provincial prefectural-city MRIOs one by one into the IPIO table based on the assumption of an identical trade coefficient of the provinces in the IPIO table. This assumption implied that trade between cities in different provinces is constrained by trade flows between provinces in the IPIO table, which are derived from detailed VAT data and thus of relatively higher quality^8^.

### The embedding process of the provincial prefectural-city MRIO tables into the IPIO table

First, we take provinces out of the IPIO table and put the provincial prefectural-city MRIO tables in the corresponding location of the IPIO table. The next key issue is to estimate the trade matrices between each prefectural city in one prefectural-city MRIO table and each prefectural city in other prefectural-city MRIO tables in the IPIO table. We adopt the identical trade coefficients of the province with other provinces to disaggregate vectors of exports to other provinces and imports from other provinces of each prefectural city, which have been estimated in Step 1, into trade matrices between each city in the prefectural-city MRIO table and each city in other provinces and municipalities in the IPIO table. After all the 27 provincial MRIOs and the two-region Chongqing tables embedded into the IPIO table (the other three direct municipalities are kept as is), a full city-level MRIO within the mainland China boundary for one year is formed with unbalanced column and row sums. Finally, the modified RAS procedure is used to balance the full prefectural-city level MRIO table, including 340 cities in mainland China. We carry out the process across all four benchmark years.

In this stage, we obtain the third set of intermediate data products, the full prefectural-city MRIOs, depicting the intermediate and final product transactions in 42 sectors among the 340 cities in mainland China. This is the first time a full city-level MRIO by firm ownership within mainland China has been constructed.

Currently, the fifth China Economic Census and the eighth China Input-Output Survey are integrated into one undertaking for the benchmark year 2023, and they are conducted in 2024. Our combined bottom-up and top-down approach outlined above provides a more consistent data reconciliation procedure for future benchmark inter-city IO table compilations based on the forthcoming integrated new economic census data.

### Step 3: Constructing non-competitive SRIOs for Hong Kong, Macao, and Taiwan, respectively

There are no officially compiled input-output tables for Hong Kong and Macao, as most of the provinces in mainland China. Most of the existing IO tables for Hong Kong or Macao are usually by-products of the development of global input-output databases such as GTAP, Eora, and OECD-ICIO^86^. To bridge the gap between users and the compilation of IO tables for HMT as a single-region SRIO that is consistent with China’s SRIO tables, this step describes in detail how to compile non-comparative IO tables for Hong Kong, Macao, and Taiwan, respectively, for 2002, 2007, 2012, and 2017 via data available from local governments and OECD.

**Fig. 7:**
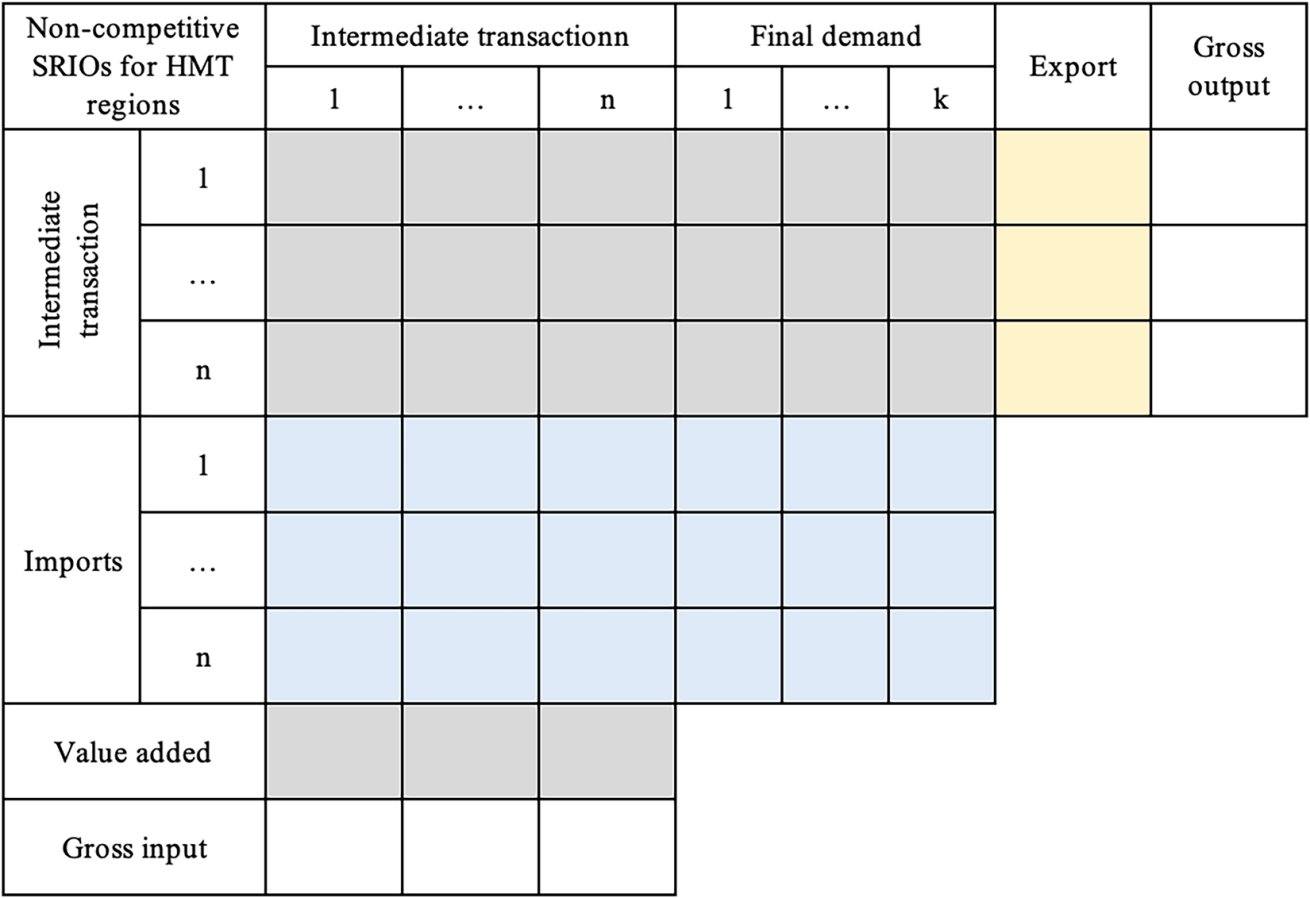
The layout of the non-competitive regional input-output table for Hong Kong, Macao, and Taiwan. The grey areas represent the domestic intermediate transaction and final demand matrices and value added vectors. The light yellow area denotes exports, and the light blue areas indicate imports from other countries.

The structure of regional tables for HMT is shown in Fig. 7. It aims to construct regional IO tables with 42 commodity sectors, five categories of final demand by sector, value added by sector, import matrix by sectors (42 by 42), and exports by sector.

### Constructing initial input-output tables of Hong Kong

Data for compiling IO tables of Hong Kong and classification conversion is taken from the OECD-ICIO database (OECD Inter-Country Input-Output Database, http://oe.cd/icio)^86^. OECD has compiled annual input-output tables for Hong Kong from 1995 to 2020, covering 45 industrial sectors. We first extract input-output tables at the basic price for Hong Kong for 2002, 2007, 2012, and 2017 from the OECD ICIO database and transfer them into producers’ prices using margin sheets of production tax minus subsidies. The industrial-sector classification of the OECD tables is based on the International Standard Industrial Classification (ISIC, revision 4), which differs from the IO-sector classifications published by the NBS of China. China’s IO-sector classifications differed slightly across benchmark years 2002, 2007, 2012 and 2017, which correspond to CSIC versions of GB/T 4754-1994, GB/T 4754-2002, GB/T 4754-2011, and GB/T 4754-2017, respectively. The concordance map is developed to harmonise OECD sector classification with China’s IO-sector classifications using Hong Kong’s gross output by sector as proxies to convert the original OECD IO tables into ones by the Chinese IO classifications.

In addition, categories of final demand in OECD IO tables are inconsistent with those of Chinese provincial/national IO tables. We combined household final consumption expenditure and non-profit institutions serving households into household consumption expenditure; direct purchases abroad by residents and imports (cross border) into imports/re-import; direct purchases by non-residents and exports (cross border) into exports/re-exports in the OECD table, respectively. This consolidates five categories of domestic final demand into four: household consumption expenditure, government consumption expenditure, gross fixed capital formation, and changes in inventories. These categories align with those used in Chinese provincial/national input-output tables.

### Constructing initial input-output tables of Macao

Since there are no officially compiled IO tables for Macao, this paper constructs Macao’s IO tables for 2002, 2007, 2012, and 2017. The main data sources for the compilation are the Statistical Yearbooks of Macao and the Statistics Database of the Government of Macao Special Administrative Region Statistics and Census Service (DSEC), which provides national accounts and industrial information, including gross output, industrial value added, major components of expenditure-side GDP, exports and imports. DSEC also provides household consumption expenditure in detail by Household Income and Expenditure Survey of Macao for the years 2002/2003, 2007/2008, 2012/2013, and 2017/2018. These key variables by sector are mapped by concordances constructed between their original classifications and the 42-IO classification in our database. The initial estimates of the intermediate transaction matrix and final demand matrix are estimated by direct input coefficients of the intermediates and the distribution structure of the final demand from Hong Kong’s IO tables, respectively. Intermediate inputs, final demand, and gross output of each sector in Macao are used as the column and row constraints.

### Constructing initial input-output tables of Taiwan

Taiwan has officially compiled competitive commodity-by-commodity IO tables at producers’ prices covering 49 commodity sectors for the year 2001, 52 sectors for 2006 and 2011, and 63 sectors for 2016, and competitive supply tables at purchasers’ prices covering 50 sectors for 2002, 2007, 2012, and 2017^87^. Taiwan also publishes separate domestic and import transaction matrices in its use tables for all four benchmark years. These IO tables are converted into 42 sectors that are consistent with mainland China’s IO-sector classifications used in our database. The conversion process across four years is consistent, and we only highlight the 2012 table as an example to illustrate the process: First, the IO table at producers’ prices with 52 sectors in 2011 is aggregated into 50 sectors, and the intermediate transaction matrix and final demand matrix are used as the initial structure. Second, the supply table at purchasers’ price with 52 sectors in 2012 is aggregated into 50 sectors, and the gross output, value added, intermediate input and intermediate output by sectors are used as the initial constraints. Third, a standard RAS procedure is applied to obtain the competitive IO table of the 50 sectors in 2012. Fourth, Taiwan’s domestic and import transaction matrices in its 2011 use table with 50 commodity sectors and 52 industry sectors are converted to 50 by 50 commodity IO tables as the basis for converting the competitive table to a non-competitive one. Finally, the non-competitive IO table is aggregated into 42 commodity sectors. The same process is carried out for the years 2002, 2007, and 2017.

### Balancing the initial input-output tables of HMT

With the initial estimations of non-competitive IO tables with 42 sectors for Hong Kong, Macao, and Taiwan in hand, the RAS procedure is applied to balance these initial non-competitive IO tables to obtain the final IO tables of Hong Kong, Macao, and Taiwan for years 2002, 2007, 2012, and 2017, respectively.

### Step 4: Linking prefectural-city MRIOs by firm ownership for mainland China and HMT SRIOs to form the full inter-city IO table for the Greater China area

**Fig. 8:**
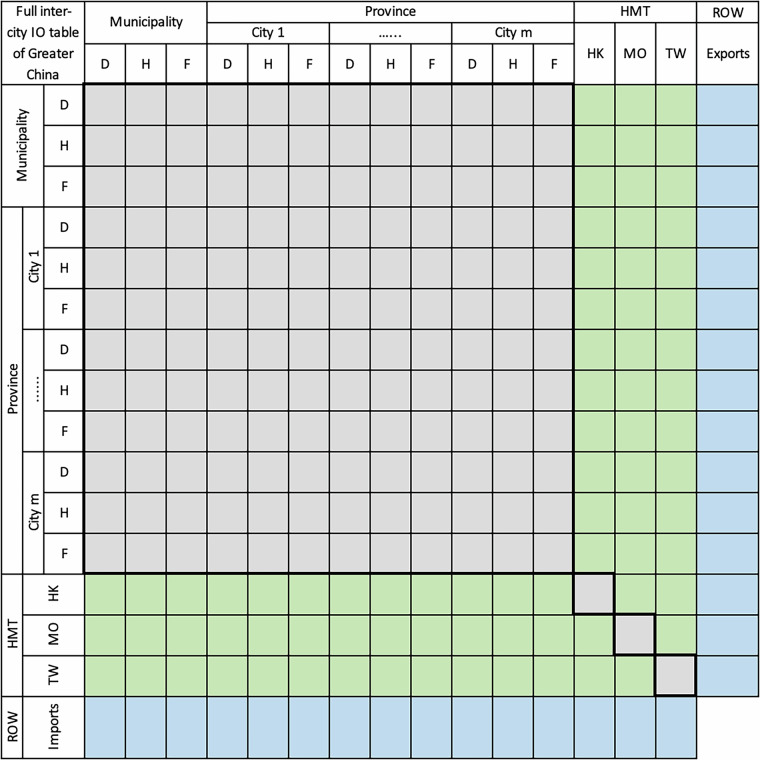
The illustration of the full inter-city IO table for the Greater China area. The grey areas represent the 340-city MRIO table for mainland China and the regional input-output tables for Hong Kong, Macao, and Taiwan. The green areas indicate inter-city trade between each city and Hong Kong, Macao, and Taiwan, respectively. The blue areas represent exports to and imports from other countries for each city, as well as for Hong Kong, Macao, and Taiwan.

This step aims to establish a more holistic and interconnected economic landscape of the Greater China area by assembling prefectural-city MRIOs by firm ownership of mainland China and three SRIO tables for Hong Kong, Macao, and Taiwan, representing the key economic areas linking mainland China and the world. As shown in Fig. 8, addressing four tables in the diagonal location of the Great China table (Fig. 8, grey areas) and estimating trade flow by sector between regions in the four tables are key steps for linking the four tables (Fig. 8, green and blue areas).

### The acquisition of bilateral trade data between mainland China, HMT regions, and the rest of the world

The detailed bilateral trade data, i.e., exports and imports by sector distinguishing intermediate use and final demand between mainland China, HMT regions and the rest of the world, have been aggregated by UN Broad Economic Category (BEC) end-use categories of consumption goods, capital goods, and intermediate goods as shown in Chen *et al*.^8^. Because of intermediary trade in Hong Kong, bilateral trade data between Hong Kong and other regions need to be adjusted. OECD has used very detailed custom data of Hong Kong and other entrepôt economies such as Singapore and the Netherlands to re-allocate Hong Kong’s re-exports and re-imports to their origin and destination regions, so we use the bilateral trade data from OECD directly. After obtaining the bilateral trade data that separates intermediate and final goods trade flows between each of these regions, the methodology proceeds to integrate these four tables as follows.

### Estimating the inter-regional intermediate and final trade matrices

Given that the exports by sector from one city to other regions have been divided into intermediate and final products, we compute the distribution coefficient matrix for the imports of intermediate and final products in another region. Such coefficients are defined as the ratio of a particular intermediate or final product import to the sum of all intermediate or final product imports by another region. The intermediate and final trade matrix from one city to another region is estimated by sector totals multiplied by the distribution coefficient matrix for intermediate and final products, respectively.

### Updating exports by sector and import matrix for each region

The updated exports by sector of one region are calculated by subtracting the sum of the inter-regional flows of intermediate and final products from the initial exports by sector. This process adjusts the exports by sector to account for the outgoing flows of commodities to other regions. Similarly, the updated import matrix is obtained by deducting the sum of inter-regional flows of commodities into the region from the initial import matrix. This step revises the import matrix to accommodate the incoming flows of commodities from other regions.

By merging the prefectural-city MRIOs of mainland China and regional input-output tables of HMT regions in the Greater China area, it aligns these matrices more closely with the actual economic interplay between the regions, considering the dynamic nature of trade flows. This iterative procedure facilitates an accurate representation of the current state of economic relations, thus enriching our understanding of the economic geography within the Greater China area.

## Data Records

The inter-city IO database presents the city-level economic structure and inter-city trade flows for three municipalities, Chongqing with two regions and 335 prefecture cities in 27 provinces in mainland China, as well as Hong Kong, Macao, and Taiwan for 2002, 2007, 2012, and 2017. Each city in mainland China includes 42 commodity sectors distinguishing three types of firm ownership, while HMT each has 42 sectors without firm type information.

Four sets of derived IO tables are used in the final complete inter-city IO table construction. The first three sets of derived IO tables featured with firm ownership are the non-competitive prefectural-city SRIO tables by firm ownership for 337 cities, the prefectural-city MRIO tables by firm ownership for 27 provinces and Chongqing, and the prefectural-city MRIO tables by firm ownership with 340 cities in mainland China. They are innovative sets of IO tables constructed for the first time, distinguishing each sector with domestically-, HMT-, and foreign-owned firms to reflect firm heterogeneity. The fourth one is non-competitive regional input-output tables without distinguishing firm ownership for Hong Kong, Macao, and Taiwan, respectively.

A non-competitive prefectural-city SRIO table by firm ownership (Fig. 4) includes an intermediate transaction matrix (126*126) for the 42 sectors by three types of firm ownership and a final demand matrix (126*5) containing 42 sectors by three types of firm ownership and five categories (urban household consumption, rural household consumption, government consumption, fixed capital formation and changes in inventories). The row vector of value added (1*126) with 42 sectors by three types of firm ownership is located at the bottom row of the SRIO table. The column vectors of exports to other cities in its province, other provinces and other countries (126*3) and gross output (126*1) with 42 sectors by three types of firm ownership are the far-right columns of the SRIO table. The import matrix from other cities in its province (42*126) and vectors of imports from other provinces and other countries (2*126) are the bottom rows of the intermediate transaction matrix.

A prefectural-city MRIO table by firm ownership for one province (Fig. 5), assuming with *m* cities in the province, includes an intermediate transaction matrix (126 m*126 m) for the 42 sectors by three types of firm ownership and a final demand matrix (126 m*5 m) containing 42 sectors by three types of firm ownership and five categories (urban household consumption, rural household consumption, government consumption, fixed capital formation and changes in inventories). The row vector of value added (1*126 m) with 42 sectors by three types of firm ownership is located at the bottom row of the SRIO table. The column vectors of exports to other provinces and other countries (126 m*2) and gross output (126 m*1) with 42 sectors by three types of firm ownership are the far-right columns of the SRIO table. Vectors of imports from other provinces and other countries (2*126 m) are the bottom row of the intermediate and final demand transaction matrix.

A prefectural-city MRIO table by firm ownership for mainland China with 340 cities (Fig. 6) includes an intermediate transaction matrix (42840*42840) for the 42 sectors by three types of firm ownership and a final demand matrix (42840*1700) containing 42 sectors by three types of firm ownership and five categories (urban household consumption, rural household consumption, government consumption, fixed capital formation and changes in inventories). The row vector of value added (1*42840) with 42 sectors by three types of firm ownership is located at the bottom row of the MRIO table. The column vector of exports to other countries (42840*1) and gross output (42840*1) with 42 sectors by three types of firm ownership are the far-right columns of the MRIO table. The vector of imports from other countries (1*42840) is at the bottom row of the intermediate and final demand transaction matrices.

A non-competitive regional input-output table for Hong Kong, Macao, and Taiwan (Fig. 7). contains an intermediate transaction matrix (42*42) for the 42 sectors and a final demand matrix (42*4) containing 42 sectors and four categories (household consumption, government consumption, fixed capital formation and changes in inventories). The row vector of value added (1*42) with 42 sectors is located at the bottom row of the table. The column vectors of exports to other countries (42*1) and gross output (42*1) with 42 sectors are far-right columns of the table. The import matrix from other countries (42*42) is at the bottom row of the intermediate and final demand matrices.

**Fig. 9:**
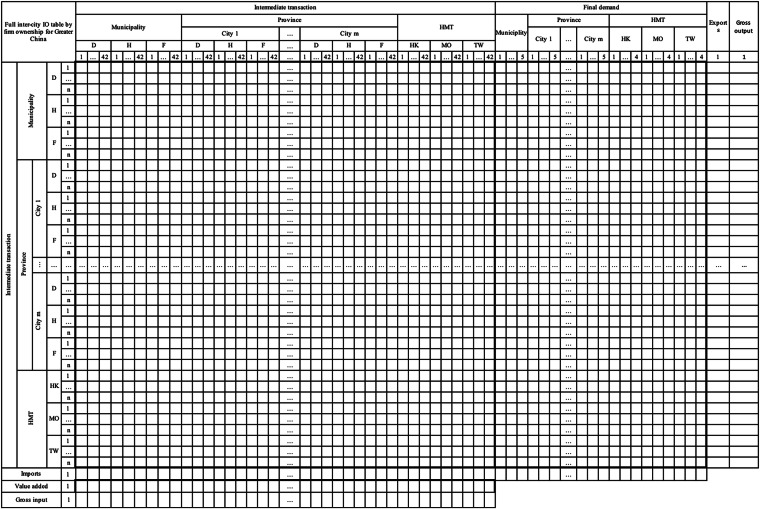
The layout of the full inter-city IO table by firm ownership for the Greater China area for one year. For simplicity, it only shows one municipality and one province as an illustration. The final table have four municipalities, of which Beijing, Tianjin, and Shanghai are regarded as city-level regions, and Chongqing is separated into two regions: urban area and rural area, 335 prefectural cities in 27 provinces, and Hong Kong, Macao, and Taiwan, totally 343 regions.

The layout of a full inter-city IO table by firm ownership for the Greater China area is illustrated in Fig. 9. For each benchmark year, the full MRIO table contains an intermediate transaction matrix (42966*42966) for the 42 sectors with three types of firm ownership in 340 cities and 42 sectors in HMT, respectively. The final demand of each region in mainland China consists of five categories, including urban household consumption, rural household consumption, government consumption, fixed capital formation and changes in inventories, while the final demand of HMT includes four categories, i.e., household consumption, government consumption, fixed capital formation and changes in inventories. The final demand matrix contains 42966$\ast$1712 vectors for each year. Also, exports to the rest of the world contain a 42966*1 vector measuring the foreign exports for all 343 regions, while imports contain 42 * 42966 vectors indicating the imports from all over the world used by the 42 sectors in each of the 343 cities. Value added is a $4$ *42966 row vectors representing value added for 340 cities and 42 sectors, distinguishing three types of firm ownership for cities in mainland China and HTM regions with 42 sectors. The total data points in the full inter-city IO table for each benchmark year are more than 1.9 billion.

The final inter-city IO tables constructed in this project can be downloaded free from the *figshare* repository. Processed data used in table compilation, include: (1) Gross output, value added and export delivery by IO sector and firm type for 335 prefectural cities in 27 provinces and two areas of Chongqing; (2) bilateral merchandise trade statistics by IO sectors and end-use categories between each of the 340 cities in mainland China and Hong Kong, Macao, Taiwan and rest of the world as well as trade statistics among HMT; and the four sets of derived IO tables, including: (1) prefectural-city SRIOs by firm ownership (total of 335 in all 27 provinces and two in Chongqing, each SRIO (170*135); (2) provincial-wide prefectural-city MRIOs by firm ownership for 27 provinces and Chongqing with urban and rural areas((126 + 4)*(131+3)); (3) the full prefectural-city MRIOs covering 340 above the prefectural level city, 42 sector and 3 type firms in mainland China(42842*44542), and (4) Non-competitive IO tables for Hong Kong, Macao, and Taiwan (86*48), are also stored in the *figshare* repository for free download^88^.

## Technical Validation

### Comparing shares of estimated value added with shares from *China City Statistical Yearbooks*

The primary goal of this subsection is to validate the sectoral value added in the initial and final database for each city. As mentioned in the Methods section, a large portion of the initial value added by sector is estimated based on the initial structure of sectoral output or employment from *Provincial Economic Census Yearbooks*, by controlling value added ratio computed from the microdata, if they are missing from city-level *Statistical Yearbooks* (see Sub-step a). In this part, we validate the proxy of the initial sectoral value added we estimated by comparing them with the ones reported by *China City Statistical Yearbooks* (note that it is different from city-level *Statistical Yearbooks* and see more details about their difference in the notes of Table 3).

**Fig. 10:**
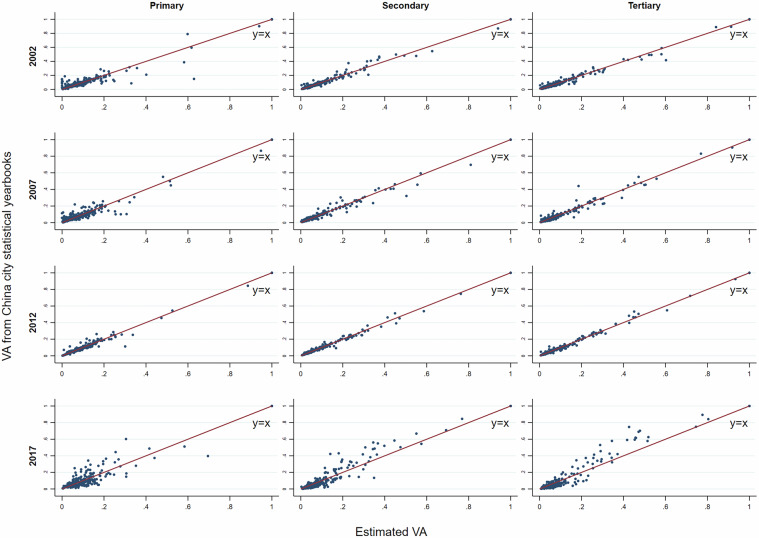
Comparing shares of estimated value added based on *Provincial* *Economic Census Yearbooks* and city-level *Statistical Yearbooks* with those from *China City Statistical Yearbooks*.

*China City Statistical Yearbooks* report the city-level value added by three major industries for most cities (about 300). We calculate the shares of reported value added for each city in all available cities within their province and compare the shares to the results based on our estimated value added. For the estimated shares of value added from *Economic Census Yearbooks* and city-level *Statistical Yearbooks*, we aggregate the sectoral value added by city into the city-level value added for the three major industries. Then, we calculated the value added contribution for each city to all available cities within the province to compare the corresponding calculated shares based on the statistics in *China City Statistical Yearbooks*. Figure 10 shows that most points fit nicely along the 45-degree lines. The comparison results of 2017 seem to show more deviation from the structure of the *China City Statistical Yearbooks* than the results for other benchmark years. This may be due to the fact that microdata from 2015, which we used as a proxy to calculate the structure for the benchmark year 2017, are somewhat outdated. However, the overall similarity between our estimates and the reported results in the *China City Statistical Yearbooks* still holds. These results demonstrate the overall similarity between our estimated value added from the *Provincial Economic Census Yearbooks* and the reported data from the *China City Statistical Yearbooks* concerning the provincial structure of value added by city for major industries. This similarity validates our estimation, which we use as the initial level of sectoral value added for each city for calibration purposes.

### Comparing the differences of the GDP of cities from our database with those from *Statistical Yearbooks*

The GDP of cities derived from city value added at the sector level from our inter-city IO tables differs from *Statistical Yearbooks* reported by local authorities. The GDP data of most cities used in this validation can be directly taken from provincial *Statistical Yearbooks*. Only a small part of cities’ GDP is obtained from city-level *Statistical Yearbooks*. The main reason for such differences is that value added in our inter-city IO tables are estimated by sector based on microdata and constrained by adjusted provincial level GDP published by NBS of China based on the economic census as described in the Methods section, while the city-level GDP for 2002, 2007, 2012, and 2017 reported by local authorities in the *Statistical Yearbooks* did not reflect the new information in the Economic Census.

The differences between the revised GDP and the GDP directly reported in *Statistical Yearbooks* at the provincial level for 2002, 2007, 2012, and 2017 are listed in Table 8. NBS of China and provincial Bureaus of Statistics revise GDP estimates following the completion of the Economic Census as more information becomes available in the Census years. That is, the revisions to the historical GDP of 1993–2004, 2005–2008, 2009–2013, and 2014–2018 are adjusted by the 2004, 2008, 2013 and 2018 Economic Census, respectively. The difference between GDP derived from our inter-city IO tables and what is reported from city-level *Statistical Yearbooks* is largely consistent *with* such adjustments. For example, the GDP reductions of Tianjin in our IO tables relative to *Statistical Yearbooks* for 2012 and 2017 are 30.40% and 32.88%, respectively, which is consistent with historical GDP revisions released by NBS adjusted based on economic census data, 29.87% in 2012 and 32.88% in 2017. The GDP of most cities in Inner Mongolia and Northeast reduced by more than 25% in 2012, except for Baishan (22.40%) in Jilin and Daqing (23.70%) in Heilongjiang. Revisions based on the 2013 Economic Census to the early estimates of GDP for 2012 in Inner Mongolia, Liaoning, Jilin, and Heilongjiang are reduced by 34.07%, 28.16%, 27.32%, and 19.54%, respectively. These reductions demonstrate the adjustments in our inter-city IO table for 2012 are very reasonable.

**Table 8:** Differences of provincial GDP between the revisions to historical GDP and Statistical Yearbooks.

Province code	Province name	2002	2007	2012	2017
11	Beijing	40.87%	11.46%	6.41%	6.67%
12	Tianjin	−6.06%	−17.66%	−29.87%	−32.88%
13	Hebei	−9.86%	−11.35%	−13.16%	−9.92%
14	Shanxi	15.23%	3.53%	−3.55%	−6.72%
15	Inner Mongolia	11.91%	−15.17%	−34.07%	−7.44%
21	Liaoning	0.00%	−6.63%	−28.16%	−7.33%
22	Jilin	−9.04%	−22.79%	−27.32%	−26.92%
23	Heilongjiang	−16.47%	−13.29%	−19.54%	−22.57%
31	Shanghai	7.14%	5.66%	5.57%	7.48%
32	Jiangsu	−0.23%	0.96%	−0.66%	0.00%
33	Zhejiang	3.14%	−0.75%	−0.82%	1.23%
34	Anhui	7.25%	7.84%	6.56%	9.84%
35	Fujian	−4.58%	0.83%	2.48%	5.16%
36	Jiangxi	0.00%	5.04%	−1.09%	1.02%
37	Shandong	−4.51%	−12.51%	−14.11%	−13.25%
41	Henan	−2.16%	−1.25%	−2.15%	0.61%
42	Hubei	−15.33%	2.39%	1.53%	4.95%
43	Hunan	−4.36%	0.93%	−4.27%	−0.22%
44	Guangdong	15.57%	2.12%	−0.11%	2.17%
45	Guangxi	2.78%	−8.07%	−13.28%	−3.95%
46	Hainan	6.38%	0.88%	−2.32%	0.78%
50	Chongqing	15.65%	15.72%	1.63%	3.30%
51	Sichuan	−3.08%	0.54%	0.21%	2.50%
52	Guizhou	4.92%	3.85%	−1.61%	0.48%
53	Yunnan	5.66%	7.09%	7.64%	12.88%
54	Tibet	0.36%	0.56%	1.31%	2.90%
61	Shaanxi	10.68%	3.95%	−2.15%	−1.94%
62	Gansu	6.08%	−1.01%	−4.55%	−1.65%
63	Qinghai	−0.12%	−8.10%	−19.28%	−6.09%
64	Ningxia	14.55%	−1.30%	−8.98%	−7.06%
65	Xinjiang	0.90%	−0.66%	−1.25%	2.55%

**Fig. 11:**
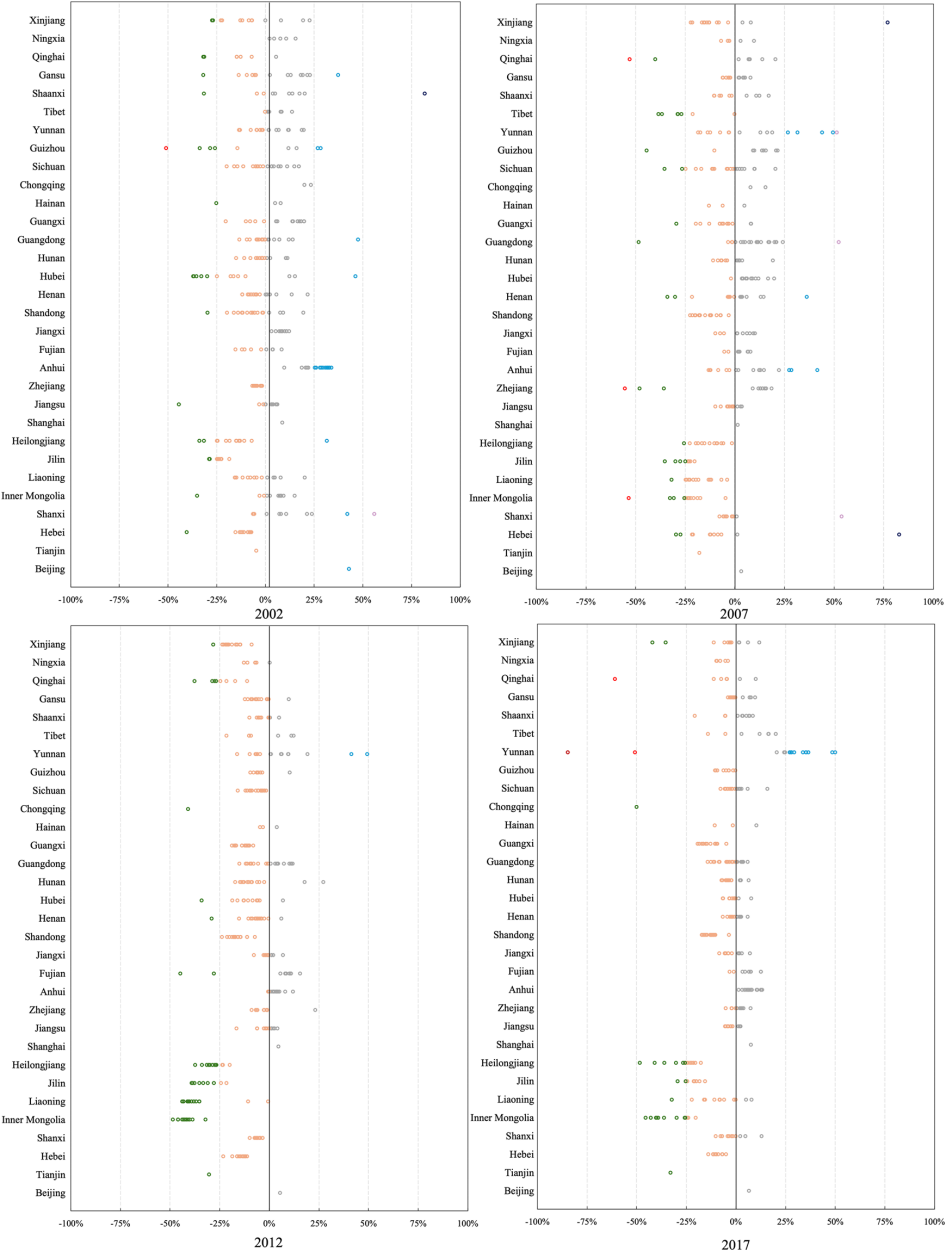
Discrepancies of the GDP of 340 cities between inter-city IO Tables by firm ownership and *Statistical Yearbooks* (by percentage %) for 2002, 2007, 2012, and 2017.

Figure 11 prints out the differences in GDP for all 340 cities arranged by province in mainland China between our database and that reported in *Statistical Yearbooks* in detail. The colours of the dots in the figure represent the discrepancy distribution in each one-quarter interval, i.e., (−100%, −75%) – dark red, (−75%, −50%) – red, (−50%, −25%) – green, (−25%, 0%) – orange, (0%, 25%) – grey, (25%, 50%) – blue, (50%, 75%) – purple, and (75%, 100%) – black. It shows that the value added at the sector level and resulting adjustment of city-level GDP in our database are basically consistent with NBS revised National Account statistics. Moreover, Fig. 11 also indicates that the differences for most cities are less than 25%, but cities spread out in 2002 and 2007 much more than in 2012 and 2017, and the GDP differences show a significant central tendency in 2012 and 2017. The GDP of Tianjin and cities in Inner Mongolia, Liaoning, Jilin, and Heilongjiang from our databased are much smaller in 2012than what local authorities reported in the provincial *Statistical Yearbooks*, thus providing solid evidence to justify the usage of microdata to adjust and estimate the economic structure at city level and validate the reliability of our data reconciliation method used in this paper. However, there are few outliers, for example, GDP of more than half of cities in Anhui in 2002 and in Yunnan in 2017 from our database are relative larger than what reported by local authorities with differences being between 25% and 50%, despite the positive adjustment is consistent with the provincial level GDP revision reported in Table 8.

### Checking the differences between the income and production-side GDP of each city

**Fig. 12:**
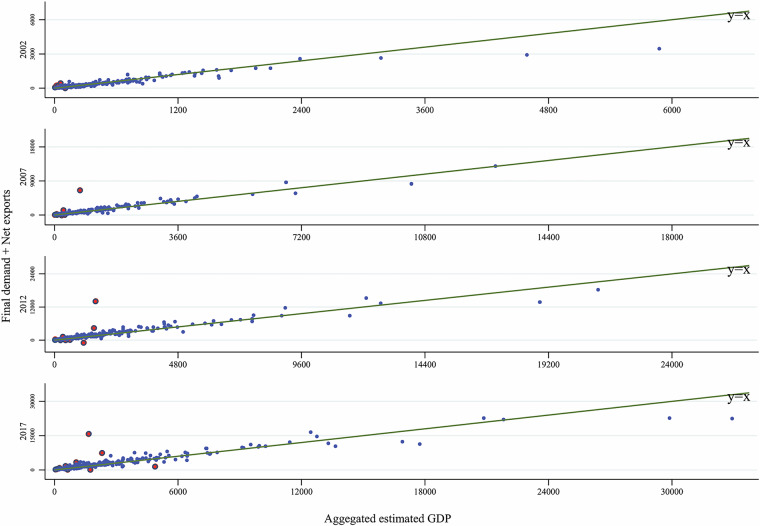
Comparing the aggregated estimated GDP calculated from production side with the final demand plus next exports. The unit of the axis is 100 million CNY. Ten cities with the largest percentage difference (i.e., $\frac{{\rm{|X}}-{\rm{Y|}}}{({\rm{X}}+{\rm{Y}})/2}$) are marked by the red circle.

As mentioned in the Methods section, the value added, i.e., GDP, calculated by the income-side approach, is directly taken from city-level *Statistical Yearbooks*, while the household consumption expenditure, estimated based on the city-level *Statistical Yearbooks* and CHIP, is from the expenditure-side GDP. Here, we check the difference between each city’s GDP calculated by the income-side approach and the expenditure-side approach, i.e., the final demand plus the net exports. The difference represents the net trade flow within China. Generally, the final demand plus the total value of net exports captures the main amount of the GDP, we thus can check the consistency between the final demand plus net exports and the GDP. Figure 12 shows how different the total factor payment in a city is from the final demand plus net exports for each benchmark year. The consistency holds for most cities except some cities, which show a large gap mainly caused by the absence of the net trade flow to other cities in China from the city’s GDP. The ten cities with the largest percentage difference in each year are listed in Table 9 (also see the red circle in Fig. 12).

**Table 9:** Summary of GDP for ten cities with the largest percentage difference.

Province Name	City Name	GDP	Final demand	Exports	Imports	Deviation
**2002**						
Tibet	Ngar	5.04	75.00	0.12	0.00	174.9%
Tibet	Lhasa	37.76	436.20	8.84	4.52	168.4%
Xinjiang	Xinjiang Other	42.52	0.00	7.07	3.01	165.1%
Tibet	Changdu	24.43	240.67	0.00	0.00	163.1%
Tibet	Linzhi	21.94	192.21	0.12	0.00	159.0%
Tibet	Naqu	15.43	132.07	0.09	0.00	158.2%
Xinjiang	Kizilsu Kirgiz	10.07	83.45	0.24	1.42	156.4%
Tibet	Shannan	23.09	159.90	0.00	0.00	149.5%
Ningxia	Guyuan	27.65	184.14	0.04	0.00	147.8%
Ningxia	Wuzhong	42.81	277.15	3.29	3.78	146.4%
**2007**						
Gansu	Jinchang	211.44	97.40	13.48	246.31	912.6%
Anhui	Tongling	294.26	219.45	24.52	221.76	171.9%
Shanxi	Xianyang	66.46	591.56	42.86	12.50	161.4%
Jilin	Jilin	702.52	6468.50	99.45	58.38	161.0%
Tibet	Ngar	7.39	64.20	0.19	0.00	158.8%
Xinjiang	Bortala	103.66	69.30	16.97	69.96	145.6%
Tibet	Shannan	21.06	124.79	0.05	0.00	142.3%
Tibet	Rikaze	34.88	199.75	0.37	0.08	140.6%
Tibet	Naqu	18.06	99.98	0.08	0.02	138.8%
Shanxi	Xinzhou	245.33	1251.27	5.77	0.62	134.7%
**2012**						
Shandong	Rizhao	1026.96	1274.03	274.84	2468.75	3635.9%
Guangxi	Hechi	436.14	479.29	36.44	502.14	187.9%
Gansu	Jinchang	216.16	219.31	3.18	215.09	186.8%
Jilin	Jilin	1730.78	14078.30	54.94	81.44	156.1%
Guangxi	Qinzhou	642.80	777.98	49.24	741.33	152.9%
Hainan	Sanya	252.63	1208.56	30.07	11.60	131.7%
Qinghai	Yushu	57.74	276.53	0.00	0.00	130.9%
Henan	Henan other	1641.57	388.24	185.69	165.73	120.3%
Tibet	Ngar	12.10	46.53	0.30	0.02	117.8%
Xinjiang	Kashgar	331.03	931.02	75.19	0.39	101.0%
**2017**						
Shandong	Rizhao	1832.99	1922.14	378.18	2214.03	182.0%
Jilin	Jilin	1897.16	15780.00	68.21	63.63	157.1%
Guangxi	Hechi	765.04	690.27	19.43	595.90	148.2%
Xinjiang	Tacheng	144.79	906.58	10.13	8.41	145.0%
Tibet	Ngar	33.01	171.89	0.01	0.01	135.6%
Xinjiang	Xinjiang Other	650.45	3281.20	29.59	6.75	134.2%
Yunnan	Diqing	119.68	445.62	1.71	0.34	115.5%
Tibet	Naqu	75.34	269.82	0.00	0.02	112.7%
Jilin	Changchun	4913.44	2656.79	140.41	1338.98	108.5%
Ningxia	Guyuan	224.43	755.56	0.01	0.12	108.4%

GDP is estimated based on city-level *Statistical Yearboo*ks and Census yearbooks. The difference is the percentage difference between GDP and the final demand plus net exports. The unit of GDP, final demand, imports, and exports is 100 million CNY.

### Consistency checking of microdata with *Statistical Yearbooks*

The key information in our database is the output, value added, and intermediate input by firm types. We mainly use microdata from *Economic Censuses* and *ASIF* to construct the shares of output by firm types to split the key variables. The challenge of using microdata to estimate the relevant shares by firm types is the inconsistency between microdata and macro data. In this section, we use the information on output from *China City Statistical Yearbooks* to check the consistency between the microdata we used, and the city-level data reported by the local statistical agencies.

*China City Statistical Yearbooks* report the output by firm types for above-scale industrial firms. We aggregate the output of the above-scale industrial firms in the microdata by firm types and compare the values reported by the *China City Statistical Yearbooks*. If the results from the two data sources are very close, the similarity between the two sources partially validates the consistency between microdata and the official city-level data for above-scale firms. Since the official data only reports the output by firm types of above-scale industrial firms, we use this comparison to display the consistency between microdata and macro data at the city level.

**Fig. 13:**
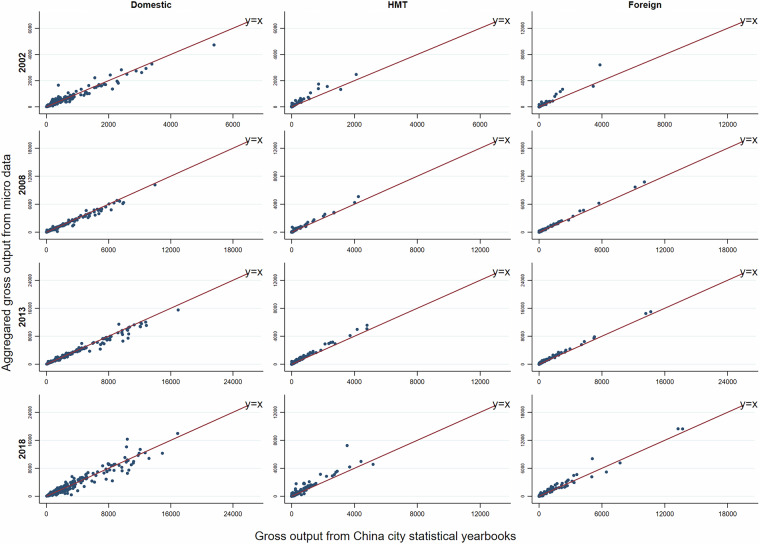
Comparing output by firm types from microdata with that from *China City Statistical Yearbooks* for 2004, 2008, 2013, and 2015. The unit of the axis is 100 million CNY. (Domestic) represents domestically-owned firms; (HMT) represents Hong Kong-, Macao-, and Taiwan-owned firms; (Foreign) represents foreign-owned firms.

Figure 13 shows the results of scatterplots by comparing microdata with the *China City Statistical Yearbooks* for years 2004, 2008, 2013, and 2015 (see the Method section for the reason for using the four years as the proxy of benchmark years). For years, using the Economic Census 2004 and 2008, we kept the above-scale industrial firms provided by NBS of China. For years, using ASIF 2013 and 2015, we aggregated all firms for comparison. Since not all cities report the output by firm types for the selected years, we only obtain part of the dots for 340 cities in our database. The numbers of cities covered in 2004, 2008, 2013, and 2015 are 276, 286, 289, and 289, respectively. More than 80% of cities are included, and most of the missing cities are small ones; the scatterplots thus can represent the overall pattern to illustrate consistency. The multi-panel figures for 2004, 2008, 2013, and 2015 demonstrate that the consistency between microdata and city-level *Statistical Yearbooks* holds except for a small proportion of cities, and most of the points in each scatterplot closely resemble a 45-degree line.

**Fig. 14:**
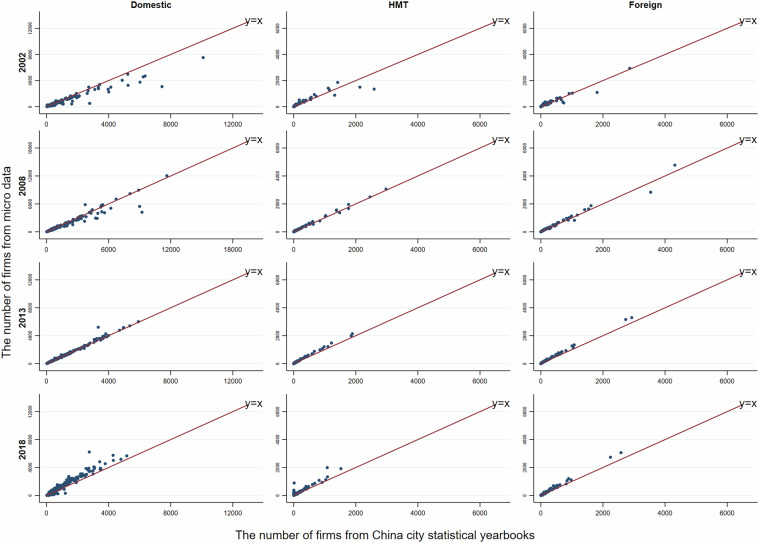
Comparing firm numbers by firm types from microdata with those from *China city Statistical Yearbooks* for 2004, 2008, 2013, and 2015. (Domestic) represents domestically-owned firms; (HMT) represents Hong Kong-, Macao-, and Taiwan-owned firms; (Foreign) represents foreign-owned firms.

In addition to comparing the output of each city’s above-scale industrial firms and its output recorded by *China City Statistical Yearbooks*, we also check the firm numbers in both sources. Figure 14 displays the comparison of firm numbers. Firm numbers show a strong linear relationship between the microdata and *China City Statistical Yearbooks*, which provides us additional confidence in the consistency of the two data sources. Also, there are more deviations and outliers in 2004 and 2015 for both output and firm numbers, implying that the difference in firm coverage in the Economic Census Database and ASIF is a possible reason for the inconsistency in estimating the shares by microdata. Besides the issue of data availability, other reasons, such as measurement errors and the location identification of a firm, can affect the consistency of using microdata to estimate city-level shares by firm types. Even though there are deviations and some outliers in the microdata, the overall pattern of the scatterplot shows a strong line relationship at a 45-degree line to confirm the consistency between microdata we used and what reported in *China City Statistical Yearbooks*.

### Compare with GBA and ADB tables for the year 2012

**Fig. 15:**
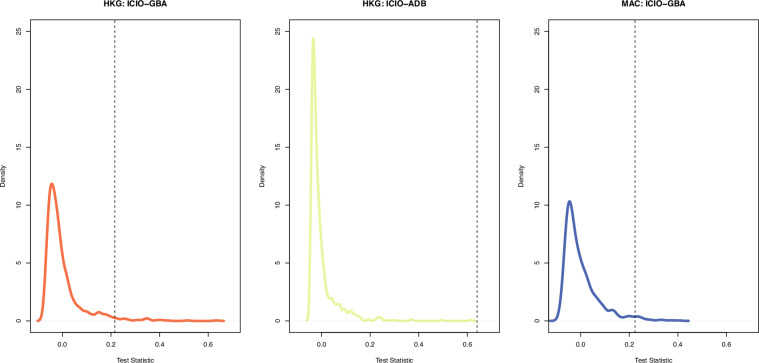
Estimated density of QAP replication with ADB HKG table and GBA HKG and Macao tables.

Asian Development Bank (ADB) publishes input-output tables for Hong Kong^88,89^ and the GBA MRIO table includes input-output tables for Hong Kong and Macao^69^. These two data sources are used for comparison with the single region IO tables of Hong Kong and Macao compiled in this study, while Taiwan is not included in this comparison because Taiwan’s IO tables come from higher quality official statistics with fewer adjustments. Because of the different numbers of sectors in these tables (35 in ADB, 26 in GBA, and 42 in our tables), we reconcile them into a 20-sector classification (see Table 10). We first calculate the mean absolute percentage error (MAPE) to compare Hong Kong and Macao with other databases in terms of value added structure. MAPE values range from 0 to 100, with smaller values indicating more remarkable similarity. The value added structure for Hong Kong is more similar to the ADB and GBA tables (see details in Table 11). Furthermore, we compare the similarity of intermediate transaction matrices, taking the inter-industry linkage characteristics into account, and use the Quadratic Assignment Procedure (QAP)^90^ to compare these matrices. Figure 15 shows that the probability of the correlation coefficients obtained from random calculations is all greater than or equal to the actual correlation coefficients, none of which rejects the original hypothesis, i.e., intermediate transaction matrices in our single region IO tables for Hong Kong and Macao have high similarity with that in the ADB and the GBA tables.

**Table 10:** The 20-sector classification in the inter-city IO table, ADB table and GBA table.

C01	Agriculture
C02	Mining and Quarrying
C03	Food & Beverages
C04	Textiles and Wearing Apparel
C05	Wood and Paper
C06	Petroleum, Chemical and Non-Metallic Mineral Products
C07	Metal Products
C08	Electrical and Machinery
C09	Transport Equipment
C10	Other Manufacturing
C11	Electricity, Gas and Water
C12	Construction
C13	Wholesale and Retail Trade
C14	Hotels and Restaurants
C15	Transport
C16	Post and Telecommunications
C17	Financial Intermediation and Business Activities
C18	Public Administration
C19	Education, Health and Other Services
C20	Others

**Table 11:** The MAPE values derived from comparisons of the value added structure between the ADB table and the GBA table (Unit: %).

Source	GBA	ADB
Hong Kong	3.39	4.11
Macao	17.77	—

### Comparison of inter-city IO tables of mainland China by firm ownership with CEADs city-level MRIO table

**Fig. 16:**
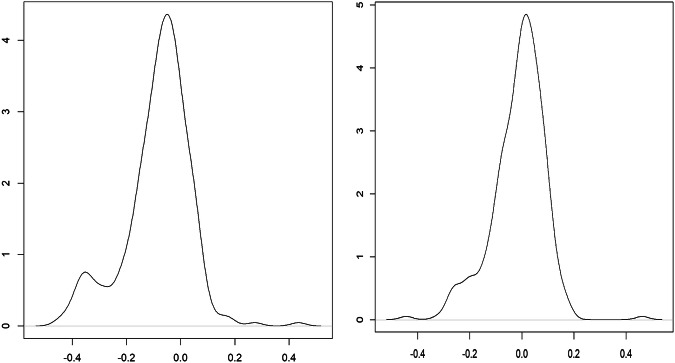
Discrepancies of value added density distribution between inter-city IO Tables by firm ownership and CEADs tables (by percentage %) for 2012 and 2017.

We closely compared our inter-city IO tables of mainland China by three types of firm ownership with Carbon Emission Accounts and Datasets (CEADs) city-level MRIO tables for China for 2012 and 2017, alongside the indicators reflecting the economic transactions within these entities. A preliminary comparative analysis of the quintessential distribution of value added at the city level is conducted based on the two distinct inter-city IO tables to encapsulate the heterogeneity intrinsic across various city-industry pairs. Figure 16 probes the effects of ownership division. We conducted a comparative analysis of the value added from our inter-city IO tables against those from the CEADs tables across cities. The mean of the discrepancy is 8.52% for 2012 and −1.34% for 2017, and the standard deviation is 12.1% for 2012 and 10.1% for 2017, implying that the value added of the two tables are close to each other in both years and had more similarities in 2017 relative to 2012. The kernel density estimation in Fig. 16 delineates the distribution of these differential values. The pronounced peak near the zero mark suggests a central tendency, with most cities/sectors pairs exhibiting a negligible difference in value added between the two sets of tables.

**Fig. 17:**
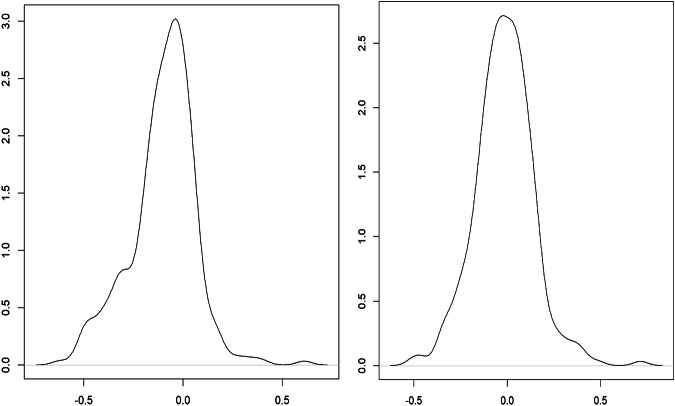
Discrepancies of value added standard deviations density distribution between inter-city IO Tables by firm ownership and CEADs tables for 2012 and 2017.

Figure 17 delineates the density distribution of relative discrepancies in sectoral value added standard deviations across cities, as represented within the two inter-city IO tables. Contrasting the within-city value added standard deviation reveals substantially higher sector-level heterogeneity when parsed by ownership in our inter-city IO tables compared to CEADs. The density distribution exhibits a pronounced positive skew, with numerous cities displaying sectoral value added markedly above the mean when partitioned by firm ownership, attesting to considerable diversity in economic structures and differential intensity of sectoral activities across urban localities.

In addition, to further verify the heterogeneity of ownership in our inter-city IO tables, we scrutinised the coefficient of variation (CV) of value added as a statistical measure of relative dispersion. On the one hand, our findings indicate that the CV for value added across diverse industries within the inter-city tables by firm ownership is significantly higher at 2.9 in 2012 and 3.0 in 2017, as compared to a CV of 1.50 in 2012 and 1.30 in 2017 in CEADs tables at the city level. This disparity aligns with theoretical expectations, attributing the increment in CV to the inherent heterogeneity introduced upon segmenting the city data by ownership categories. The nuanced differences between domestically-, HMT-, and foreign-owned firms, for instance, are likely to be manifested in the variability of their economic output.

**Fig. 18:**
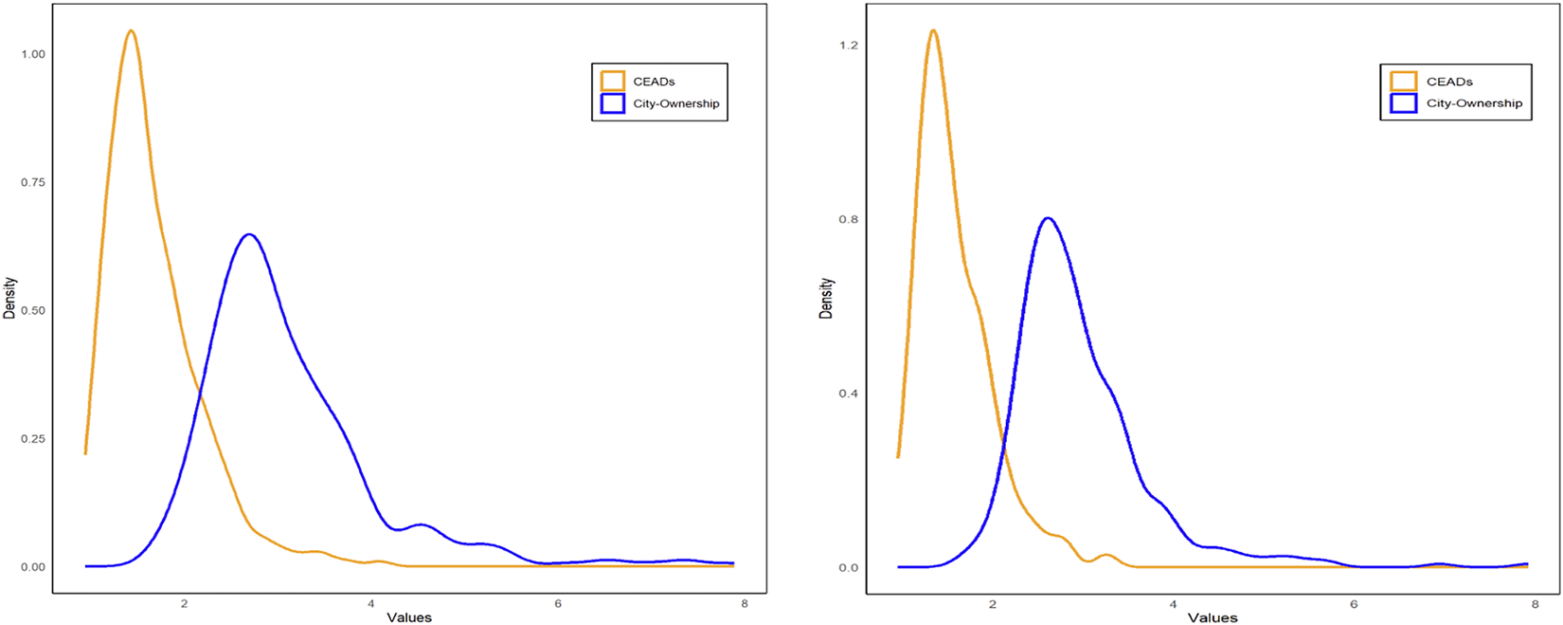
Coefficients of variation (CV) density distribution of inter-city IO tables by firm ownership and CEADs tables for 2012 and 2017.

On the other hand, a stark contrast in the distribution of value added CV values across cities emerges when comparing our inter-city IO tables and the CEADs tables, as depicted in Fig. 18. Our city-level tables, which partition data by ownership, exhibit markedly higher CV magnitudes than the CEADs. This divergence points to enhanced heterogeneity in economic production and industrial operations between urban areas when scrutinised via the ownership-disaggregated framework. The heightened CV variation signifies those analyses performed through the detailed lens, accounting for ownership stratification, capture greater diversity in the scale, composition, and dynamics of economic.

This study constructs a comprehensive inter-city IO database for the Greater China area that provides a detailed examination of economic interactions across 335 prefectural cities, four municipalities, and Hong Kong, Macao, and Taiwan. By distinguishing firm ownership of each city sector across four benchmark years, 2002, 2007, 2012, and 2017, the database provides a large-scale high-resolution economic structure with firm heterogeneity that no other MRIO tables for China in the literature with comparable richness. The innovative micro and macro data reconciliation approach (a combination approach of bottom-up and top-down methods) and a novel three-tier data architecture, enhance its accuracy and applicability, enabling in-depth analysis of inter-city connection, regional development, and firm dynamics. This database serves as a valuable tool for studying socioeconomic and interdisciplinary issues at the city level and offers insights for constructing similar inter-city IO tables in other large economies.

Despite our inter-city IO database has adopted several methodological innovations and introduced some new features, there are still limitations, especially in relation to inter-city trade or exports/imports with other countries, which can affect the accuracy of its applications such as estimating emission transfers from City A to City B. First, the uncertainty involved in inter-city trade flows remains a challenge. Due to the lack of data of inter-city trade flow, we estimate it mainly relied on the maximum entropy method, which assumes that sectoral inter-city trade is proportional to total inter-city exports and imports and inversely proportional to transport costs. Transport costs for services are assumed to be zero, potentially leading to inaccuracies in tracking actual goods and service flows between cities. Second, limitations in international trade data present additional challenges. Sectoral estimates of cities’ exports and imports from foreign countries are derived from Custom Trade Statistics provided by China’s customs authorities, which only cover trade in goods without tarde in services. As a result, capturing service-based emissions is difficult, leading to the potential underestimation of emissions embedded in service-related transactions. Finally, aggregation bias (product-mix problem) still exists when we aggregated firm-level data into city and sectoral levels, even though we have disaggregated each city/sector pair by three types of firm ownerships, the firm-level heterogeneities in production techniques and emission intensities are still not fully captured. Thus, differences in energy efficiency, technology use, and resource allocation across firms within the same sector among cities can distort emission transfer estimates. These limitations should be carefully considered when using the database for inter-city trade and environmental footprint analysis.

## Usage Notes

### The city list determination in our database

A list of cities common in the Economic Census 2008 and *Custom Trade Statistics* has been developed. On one hand, 2865 county-level 6-digit codes can be extracted from Economic Census 2008. We only take the first 4-digit codes representing prefecture-level city identification out of the 6-digit codes for the location of census firms. This lists 344 unique prefecture-level 4-digit codes for Economic Census 2008. On the other hand, to obtain more comprehensive samples for *Custom Trade Statistics*, we merged trade data from 2007–2009 and got 563 unique prefecture-level 4-digit codes after removing duplicates. Thus, in the first concordance we developed, we allocate the 2865 county-level 6-digit codes from Economic Census 2008 into 344 prefecture-level 4-digit codes from Economic Census 2008 and 563 prefecture-level 4-digit codes from *Custom Trade Statistics* to figure out how many cities are consistent in both databases. We followed the instructions of the NBS of China to determine final prefecture codes for counties. We found several prefecture-level codes have been modified due to changes in the administration district of China. We revised the 4-digit codes in custom data based on the description of the location code provided by China *Customs*. Based on this concordance, we determine the list of cities compatible with census and trade codes and divide Chongqing into ‘urban’ and ‘rural’ areas. In addition, ‘xx Other’ has been set up for the rest of the 27 provinces (in our first version of the city list). However, after further processing the census data, we found that some observations ranked into ‘xx Other’ have no real production data. Thus, except for Henan, Hainan, Hubei and Xinjiang, ‘xx Other’ for other provinces have been dropped in the 2nd version of the city list (also called ‘IO city’), which contains 340 above prefectural level cities if excluding Hong Kong, Macao, and Taiwan.

### Concordance across the province-prefecture-county level

We developed a concordance for the Economic Census 2004 and 2008 county code across the province-prefecture-county level. First, we obtain the full sample (4525 in total) of 6-digit district and county codes of Economic Census 2004 and 2008. Then, following the instructions of the NBS of China, the 4-digit codes at the prefectural level and 2-digit codes at the provincial level were compiled simultaneously. Drop the ‘xx other’ 4-digit codes except for Henan, Hainan, Hubei, and Xinjiang to keep consistency with the IO city list. After several rounds of double check, 4454 codes of 6-digit district and county codes have been included in the table. Furthermore, we expanded the sample year to cover all of the county codes for the *Economic Census*, where 58 extra county codes have been included, making 4512 county codes incorporated in the expanded concordance across the province-prefecture-county level for China.

### Concordance for location code in trade data with IO city list

We developed a concordance for location code extracted from trade data between 1995–2017 to keep consistent with the IO city list. There are 598 location codes in total. We first inquired about the corresponding city information one by one, following the instructions of China Customs staff. Thirty-nine out of these codes are missing in the instruction of *Custom Trade Statistics*, and this was finally investigated with the help of the NBS of China. It is worth noting that some 4-digit location codes end as ‘xx90’ or ‘xx91’ (which denotes the other of xx province) have been classified into the provincial capital city (xx01) with the consideration of stability of the data volume as well as comparability and consistency between years given most ‘xx Other’ have been dropped in the city list. In particular, some prefectures need special treatment due to adjustment of administrative division in China over the years, where Yanbei had been split into Datong and Shuozhou with proportion at 7/13 and 6/13 respectively; Exi as a previous prefecture including Xiangyang, Yichang, Jingzhou, Jingmen, Shiyan, Suizhou, Enshi and Shennongjia, of which the administrative name changed to Enshi with the scope remaining unchanged, has been attributed into Hubei Other; Gui’an as a state-level new district approved in 2014, located at the junction of Guiyang and Anshun, however, its economic statistics account belongs to Guiyang.

### Other concordances

Detailed concordance tables were used to reconcile different classifications of trade data from *China Custom Trade Statistics*, microdata from the Economic Census Database and AISF to China’s IO sectors, China System of Standard Industry Classification(CSIC) to China’s IO sectors, and HS code to IO sectors are the same to what has been used in the IPIO database distinguishing firm ownership for China, including HS to BEC and China’s IO sectors, CSIC to China’s IO sectors, and chained IO sector concordance among the four benchmark years. In addition, concordance tables for mapping eight major household consumption expenditures and China’s IO sectors, concordance tables for mapping OECD-ICIO and China’s IO sectors, Macao industrial sectors and China’s IO sectors, and Taiwan’s IO sectors and China’s IO sectors are made in this project.

All concordance tables described above are stored in the *figshare* repository for free download and can be used in other China-related data projects for the world research community.

## Supplementary information

Supplementary Table 1

Supplementary Table 2

## Data Availability

The computer codes used in our data processing, data generation and balance procedures are based on MATLAB, STATA and GAMS. The code used to process firm-level microdata and trade statistics is the same as Chen *et al*.^8^. The code to process household survey microdata is based on MATLAB. All raw data, derived (MR)IO tables, and the final inter-city IO tables elaborated in this work and associated codes are available in the *figshare* repository.
